# Cisplatin or LA-12 enhance killing effects of TRAIL in prostate cancer cells through Bid-dependent stimulation of mitochondrial apoptotic pathway but not caspase-10

**DOI:** 10.1371/journal.pone.0188584

**Published:** 2017-11-28

**Authors:** Olga Vondálová Blanářová, Barbora Šafaříková, Jarmila Herůdková, Martin Krkoška, Silvie Tománková, Zuzana Kahounová, Ladislav Anděra, Jan Bouchal, Gvantsa Kharaishvili, Milan Král, Petr Sova, Alois Kozubík, Alena Hyršlová Vaculová

**Affiliations:** 1 Department of Cytokinetics, Institute of Biophysics, Czech Academy of Sciences, v.v.i., Brno, Czech Republic; 2 Department of Animal Physiology and Immunology, Institute of Experimental Biology, Faculty of Science, Masaryk University, Brno, Czech Republic; 3 Center of Biomolecular and Cellular Engineering, International Clinical Research Center, St. Anne's University Hospital, Brno, Czech Republic; 4 Institute of Biotechnology, Czech Academy of Sciences, v.v.i., Praha, Czech Republic; 5 Department of Clinical and Molecular Pathology and Institute of Molecular and Translational Medicine, Faculty of Medicine and Dentistry, Palacky University, Olomouc, Czech Republic; 6 Department of Urology, Faculty of Medicine and Dentistry, Palacky University, Olomouc, Czech Republic; 7 Platinum Pharmaceuticals, a.s., Brno, Czech Republic; Universite du Quebec a Trois-Rivieres, CANADA

## Abstract

Searching for new strategies for effective elimination of human prostate cancer cells, we investigated the cooperative cytotoxic action of tumor necrosis factor-related apoptosis-inducing ligand (TRAIL) and two platinum-based complexes, cisplatin or LA-12, and related molecular mechanisms. We demonstrated a notable ability of cisplatin or LA-12 to enhance the sensitivity of several human prostate cancer cell lines to TRAIL-induced cell death via an engagement of mitochondrial apoptotic pathway. This was accompanied by augmented Bid cleavage, Bak activation, loss of mitochondrial membrane potential, activation of caspase-8, -10, -9, and -3, and XIAP cleavage. RNAi-mediated silencing of Bid or Bak in Bax-deficient DU 145 cells suppressed the drug combination-induced cytotoxicity, further underscoring the involvement of mitochondrial signaling. The caspase-10 was dispensable for enhancement of cisplatin/LA-12 and TRAIL combination-induced cell death and stimulation of Bid cleavage. Importantly, we newly demonstrated LA-12-mediated enhancement of TRAIL-induced cell death in cancer cells derived from human patient prostate tumor specimens. Our results provide convincing evidence that employing TRAIL combined with cisplatin/LA-12 could contribute to more effective killing of prostate cancer cells compared to the individual action of the drugs, and offer new mechanistic insights into their cooperative anticancer action.

## Introduction

Prostate cancer is the second most frequently diagnosed cancer and one of the leading causes of cancer deaths in men worldwide [1]. Currently available treatments mainly involve surgery, radiation therapy, hormonal therapy (androgen ablation) or chemotherapy [2]. As prostate cancer cells often develop the ability to grow in the absence of androgens or become resistant to chemotherapy, there is still no efficient cure for this type of disease especially in the later metastatic stages. Considerable attention has therefore been paid to novel tumor-selective anticancer agents whose cytotoxic potential may not strictly depend on cellular status of androgen receptor or frequently mutated p53.

The tumor necrosis factor-related apoptosis-inducing ligand (TRAIL) cytokine possesses a unique capacity to kill selectively cancer cells *in vitro* and *in vivo* without causing toxicity to normal cells or tissues [3–5]. TRAIL can trigger apoptosis by interaction with two of his five known receptors–death receptor 4 and 5 (DR4/DR5) at the cell surface. Upon its binding, DR4 and DR5 are trimerized and death-inducing signaling complex (DISC) is formed. Intracellular part of DR called death domain (DD) recruits Fas-associated death domain (FADD) protein that consequently binds initiator pro-caspase-8/-10 via the death effector domain (DED) interaction. The caspase-8 activated at the DISC further mediates effector caspase-3 activation, followed by execution of apoptotic program. Apoptotic signaling can also be enhanced by initiator caspase-mediated BH3-only protein Bid cleavage, generating truncated Bid (tBid). The tBid activates pro-apoptotic Bcl-2-family members Bak or Bax, leading to mitochondrial outer membrane permeabilization [6, 7]. Other mitochondria-related proapoptotic events such as release of cytochrome c, Smac/DIABLO, apoptosome formation, caspase-9 activation and effector caspases cleavage further multiply apoptotic death signaling [8]. Although application of recombinant TRAIL or agonistic DR4/5 monoclonal antibodies emerged as a promising anticancer strategy [9], apparent resistance of mainly primary tumors including prostate to their killing effects poses a serious obstacle in establishing clinically efficient TRAIL-based monotherapies [10, 11]. This could be overcome by combining DR4/5 ligands with some chemotherapeutic drugs.

Cisplatin is a largely employed platinum(II) compound that exerts clinical activity against numerous solid tumors, and was also shown to have potential in management of metastatic castration-resistant prostate cancer [12, 13]. However, its application may be limited due to the undesired side effects or the resistance in various cancer cell types [14, 15]. These limitations evoke a need to uncover yet unknown molecular mechanisms of cisplatin action or research and development of new platinum-based complexes with improved antitumor potential. The promising anticancer effects of platinum (IV) complex LA-12 [16] have been reported *in vitro* and *in vivo* by us and others in various cancer cell types including prostate [17–25]. In addition to their individual potential to affect cancer cell death on their own, platinum-based drugs have been suggested to sensitize prostate cancer cells to TRAIL-induced apoptosis. To date, only a limited number of studies report on the cooperative cytotoxic effects of cisplatin and TRAIL/anti-DR5 antibodies in this type of cancer [25–27]. Previously, we were the first to demonstrate the ability of LA-12 to enhance TRAIL-induced apoptosis [25], which is so far the only publication referring to the effects of the combined action of the two drugs in prostate cancer cells. The molecular mechanisms behind the above-mentioned effects are still incompletely understood.

In this study, we showed that exposure of human prostate cancer cells to sub-lethal doses of cisplatin or LA-12 resulted in profound potentiation of TRAIL-induced cell death that involved activation of mitochondrial apoptotic pathway, and investigated the novel molecular mechanisms behind the cooperative action of the drugs. We also newly demonstrated the ability of LA-12 to stimulate TRAIL-induced cytotoxicity in primary cancer cells obtained from human patient prostate tumor specimens.

## Materials and methods

### Cell culture and treatments

DU 145 prostate cancer cells derived from brain metastasis (HTB-81^TM^, ATCC, LGC Standards, Lomianki, Poland, obtained in 2014) and LNCaP prostate cancer cells derived from lymph node metastasis (#ACC 256, DSMZ Gmbh, Braunschweig, Germany, obtained in 2007) were cultured in RPMI 1640 medium (Gibco, Thermo Fisher Scientific, USA) supplemented with penicillin (100 U/ml) and streptomycin (0.1 mg/ml) (both Duchefa Biochemie B. V., Haarlem, The Netherlands) and 10% fetal bovine serum (FBS, Gibco, Thermo Fisher Scientific). PC-3 prostate bone metastatic cancer cell line (CRL-1435^TM^, ATCC, 2011) was maintained in F12 medium (Gibco, Thermo Fisher Scientific), supplemented with penicillin (100 U/ml) and streptomycin (0.1 mg/ml) (both Duchefa Biochemie B. V.) and 10% FBS (Gibco, Thermo Fisher Scientific). The cell line authentication was performed based on STR analysis (Generi Biotech, Hradec Kralove, Czech Republic) in April 2017.

Primary prostate cancer cells (PY001, PY002, PY005) were obtained from tumors (Gleason score 7; N0; M0; pT3a, pT3b, pT2c, respectively) of patients (aged 61, 71, 76 years, respectively) undergoing radical prostatectomy, following their informed written consent prior to surgery. The study was approved by the ethics committee of the Faculty of Medicine and Dentistry, Palacky University, Olomouc, Czech Republic. General derivation of PY001 is described elsewhere [28]. PY002 prostate biopsy was thawed, cut into small pieces and digested in digestion medium DMEM (Gibco, Thermo Fisher Scientific), 10% FBS (Gibco, Thermo Fisher Scientific), penicillin/streptomycin, collagenase type I (100 U/ml, Worthington, Lakewood, NJ) and dispase II (0.6 U/ml, Roche, Basel, Switzerland) for 4 hours at 37°C on rotator. The cells were then washed with DMEM with 10% FBS and penicillin/streptomycin, centrifuged (300g, 5 min), and incubated (15 min) in DMEM, 10% FBS, penicillin/streptomycin and DNase (500 U). After incubation, suspension was filtered through 70 μm filter, centrifuged (300g, 5 min), and resuspended in culture medium (see below). PY005 cells were obtained from prostate biopsy, which was directly digested using papain and trypsin, and frozen. When thawed, the cells were washed with DMEM, 10% FBS, and penicillin/streptomycin, centrifuged (300g, 5 min) and resuspended in the culture medium. The PY001, PY002 and PY005 prostate cancer cells were maintained on feeder of NIH/3T3-L1 mouse embryonic fibroblasts (kind gift from V. Bryja), which were irradiated (30 Gy), and cultivated in high glucose DMEM medium (Gibco, Thermo Fisher Scientific) supplemented with penicillin/streptomycin and 10% FBS (PAA, GE Healthcare) on plastic dishes coated with 0.02% gelatin (G2500, Sigma-Aldrich, Prague, Czech Republic) in phosphate buffer saline (PBS). The prostate cancer cells were cultivated in complete F medium [3:1 (v/v) F-12 Nutrient Mixture (Ham)/DMEM (Gibco, Thermo Fisher Scientific), 5% FBS (PAA, GE Healthcare), 0.4 μg/ml hydrocortisone, 5 μg/ml insulin, 10 ng/ml epidermal growth factor, 24 μg/ml adenine (all from Sigma–Aldrich), and 8.4 ng/ml cholera toxin (Calbiochem-MERCK, Prague, Czech Republic)] with addition of ROCK inhibitor Y-27632 dihydrochloride (5 μM, Santa Cruz Biotechnology, Santa Cruz, CA), according to the method of Liu et al. [29]. The cells isolated from the patient biopsies and used for the specific treatments were positive for cytokeratine 18 (Western blotting), confirming their epithelial nature.

All cells were cultivated in TPP plastic dishes (Trasadingen, Switzerland), maintained in a humidified atmosphere of 5% CO_2_ at 37°C and sub-cultured twice a week after exposure to EDTA/trypsin solution. The cells were treated with cisplatin [cis-diamminedichloroplatinum(II)] (Sigma–Aldrich), LA-12 [(OC-6-43)-bis (acetato)(1-adamantylamine)ammine dichloroplatinum(IV)] (Platinum Pharmaceuticals, a.s., Brno, Czech Republic), and TRAIL (human recombinant Killer TRAIL, Apronex, Prague, Czech Republic), diluted in buffer containing 20 mM HEPES, 300 mM NaCl, 0.01% Tween 20, 1% sucrose, and 1 mM 1,4-dithiotreitol (DTT), or caspase-9 inhibitor z-LEHD-fmk (20 μM in DMSO, #550381, BD Bioscience). All substances or vehicles—dimethyl sulfoxide (DMSO, Sigma-Aldrich) or appropriate buffer—were applied at the time points and concentrations indicated in the Figure legends.

### Immunoblotting analysis

The cells were harvested, whole cell lysates were prepared, separated by gel electrophoresis and Western blot analysis was performed as described previously [19]. Immunodetection was carried out with the following primary antibodies: monoclonal mouse anti-Noxa (1:500, OP180, Calbiochem-MERCK) raised against a recombinant NOXA protein, monoclonal mouse anti-Bcl-2 (1:1000, #509) raised against a synthetic peptide corresponding to amino acids 41–54 of human Bcl-2, polyclonal rabbit anti-poly(ADP-ribose) polymerase (PARP) (1:1000, #7150) against amino acids 764–1014 mapping at the C-terminus of PARP-1 of human origin (both from Santa Cruz Biotechnology), monoclonal mouse anti-caspase-8 (1:1000, #9746) against a synthetic peptide corresponding to the carboxy-terminal sequence of the p18 fragment of human caspase-8, polyclonal rabbit anti-cleaved caspase-3 (1:500, #9661) against a synthetic peptide corresponding to amino-terminal residues adjacent to (Asp175) in human caspase-3, polyclonal rabbit anti-cleaved caspase-9 (1:500, #9505) against a synthetic peptide corresponding to residues surrounding Asp315 of human caspase-9, polyclonal rabbit anti-cleaved PARP (1:1000, #9541) against a synthetic peptide corresponding to carboxy-terminal residues surrounding Asp214 in human PARP, polyclonal rabbit anti-Bak (1:1000, #3792) against a synthetic peptide corresponding to the amino-terminal residues of human Bak, polyclonal rabbit anti-Bid (1:1000, #2002) against a synthetic peptide corresponding to residues surrounding the cleavage site of human BID, monoclonal rabbit anti-Bim (1:1000, #2933) against a synthetic peptide corresponding to residues surrounding Pro25 of Bim, polyclonal rabbit anti-XIAP (1:1000, #2042) against a synthetic peptide corresponding to the amino terminus of human XIAP, polyclonal rabbit anti-Bcl-_xL_ (1:1000, #2762) against a synthetic peptide corresponding to residues surrounding Asp61 of human Bcl-_xL_, (all from Cell Signaling Technology, Danvers, MA), and monoclonal mouse anti-caspase-10 (1:500, #MO59-3, MBL, Leuven, Belgium) against a recombinant full length human caspase-10. The proteins were recognized using horseradish peroxidase-labelled secondary antibodies: anti-mouse IgG (1:3000, #NA931) or anti-rabbit IgG (1:3000, #NA934) (Amersham Biosciences, Bucks, UK), and Immobilon Western Chemiluminescent HRP Substrate (Millipore Corp, Darmstadt, Germany). An equal loading was verified by anti-β-actin antibody raised against slightly modified β-cytoplasmic actin N-terminal peptide, Ac-Asp-Asp-Asp-Ile-Ala-Ala-Leu-Val-Ile-Asp-Asn-Gly-Ser-Gly-Lys, conjugated to KLH (1:5000, #A5441, Sigma–Aldrich).

### Densitometry analysis

Following the Western blot/immunodetection, the results were subjected to densitometry analysis using ImageJ software (NIH), and represent arbitrary units relative to control (corrected by β-actin). The statistical significance (P < 0.05) was determined by one-way ANOVA followed by Fisher’s Least Significant Difference (LSD) test using STATISTICA 12 (Stat Soft, Inc., Tulsa, OK, USA) software.

### Annexin V/propidium iodide assay

The cells were harvested, washed with PBS, incubated with annexin V-fluorescein (1:200, P030-F100, Apronex, Prague, Czech Republic) in specific binding buffer for 30 min in 37°C, and then stained (5 min) with propidium iodide (PI, 0.5 μg/ml, Sigma–Aldrich). Samples were measured by flow cytometry (FACSVerse^TM^), and analyzed using FACSSuite^TM^ software version 1.0.5 (both Becton Dickinson, San Jose, CA). Minimum of 10 000 cells excluding doublets and debris were subjected to analysis and total of annexin V^+^/PI^-^ and annexin V^+^/PI^+^ cells were assessed as percentage of dead cells.

### Measurement of mitochondrial membrane potential (MMP)

The cells were incubated (20 min) in Hanks' balanced salt solution (HBSS) with 10 nM of tetramethylrhodamine ethyl ester perchlorate (TMRE, Molecular Probes, Eugene, OR, USA), then washed twice with HBSS, and analyzed by flow cytometer (FACSVerse^TM^) and FACSSuite^TM^ v1.0.5 (both Becton Dickinson). Minimum of 10 000 cells excluding doublets and debris were subjected to analysis, and the percentage of cells with decreased MMP was determined.

### Detection of caspase-3 activity

The cells were harvested, washed in PBS, fixed and stained using FITC active caspase-3 apoptosis kit (#550480, BD Pharmingen) according to the manufacturer's protocol. The percentage of cells with active caspase-3 was detected by flow cytometry (FACSVerse^TM^, FACSuite^TM^ v1.0.5).

### Analysis of Bak activation

The cells were washed with PBS, fixed (10 min, 4°C) in 1% paraformaldehyde (pH 7.4), and stained with monoclonal mouse anti-Bak antibody (1:250, #AM03, Calbiochem-MERCK) raised against recombinant human Bak from which the C-terminal and transmembrane domains have been deleted. The protocol was previously described in [30]. The analysis was performed using flow cytometry (FACSCalibur^TM^) and CellQuest software (both Becton Dickinson), and the results were expressed as percentage of cells with active Bak.

### Cell transfection and siRNA

DU 145 cells were seeded at a density of 40 000 cells/cm^2^ and cultured overnight in media without antibiotics. Transfections were carried out in Opti-MEM medium (Gibco, Thermo Fisher Scientific) with Lipofectamine 2000 or Lipofectamine RNAiMAX (both Invitrogen, Thermo Fisher Scientific) using ON-TARGETplus SMART Pool siRNA BAK1 or ON-TARGETplus Non-targeting Pool (both 25 nM, Dharmacon, Lafayette, CO); caspase-10 siRNA sense 5′-CAUUGGUAUUCCAGUUCAGdTdT-3′, antisense 5′-CUGAACUGGAAUACCAAUGdTdT-3′or non-targeting luciferase GL2 control siRNA sense 5′-CGUACGCGGAAUACUUCGAdTdT-3′, anti-sense 5′-UCGAAGUAUUCCGCGUACGdTdT-3′ (both 50 nM, Sigma-Aldrich) [31, 32]. After 4 h incubation with transfection mix, RPMI medium with 10% FBS was added, cells were cultivated overnight, then the mix was replaced by fresh complete media, and the cells were treated as indicated in Figure legends.

### Cell transduction and shRNA

Recombinant human Bid shRNA-expressing validated lentivirus (Sigma-Aldrich, TRCN0000062709, CCGGCTTTCACACAACAGTGAATTTCTCGAGAAATTCACTGTTGTGTGAAAGTTTTTG) was prepared in HEK293 TN17 cells, concentrated using PEG-It reagent (System Biosciences), resuspended in PBS and stored until use at -80°C. DU 145 cells were transduced at MOI 2, selected using puromycin (2 μg/ml), analyzed and subsequently used in experiments.

### Statistical analysis

The results of at least three independent experiments were expressed as the means + S.E.M. Statistical significance (P < 0.05) was determined by t-test or one-way ANOVA followed by Fisher’s Least Significant Difference (LSD) test using STATISTICA 12 (Stat Soft, Inc., Tulsa, OK, USA) software. The results from Western blotting are representatives of at least three independent experiments.

## Results

### Cisplatin or LA-12 enhance the sensitivity of human prostate cancer cells to TRAIL-induced cell death

**Fig 1:**
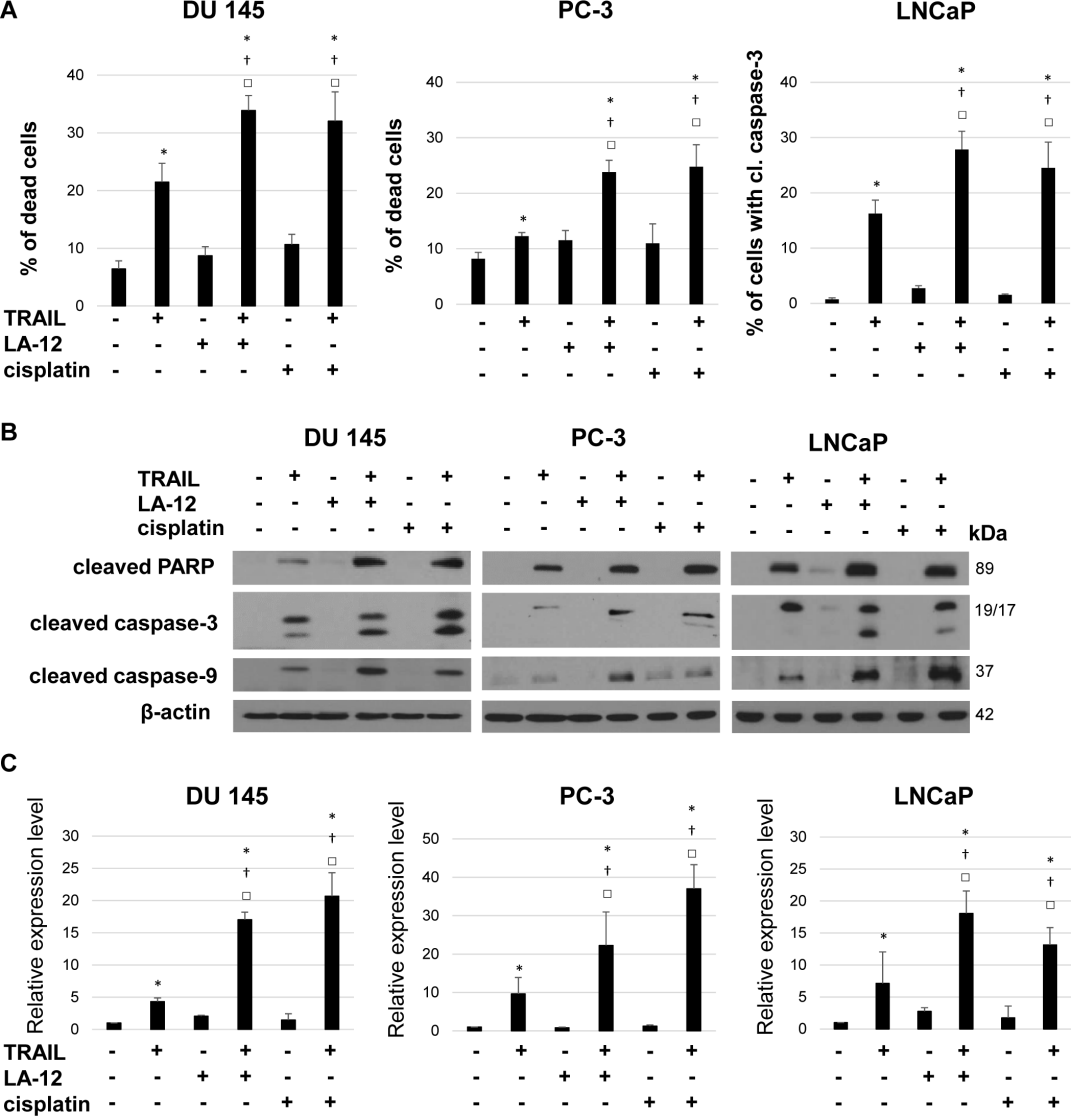
LA-12/cisplatin-mediated sensitization of DU 145, PC-3 and LNCaP prostate cancer cells to TRAIL-induced cell death. (**A**) Percentage of dead DU 145 and PC-3 cells (annexin V^+^/PI^-^ and annexin V^+^/PI^+^, flow cytometry) and percentage of LNCaP cells with cleaved caspase-3 (anti-cleaved caspase-3 antibody, flow cytometry) after their pretreatment (24 h) with LA-12 (2.5/0.5/2.5 μM) or cisplatin (5/10/5 μM) and subsequent treatment (4 h) with TRAIL (5/5/20 ng/ml), respectively (DU 145/PC-3/LNCaP). (**B**) Cleavage of PARP, caspase-3 and caspase-9 in DU 145, PC-3 and LNCaP cells treated as in (A), detected by Western blotting. The β-actin served as a loading control. (**C**) Densitometry analysis of PARP cleavage presented in (B). Numbers represent arbitrary units relative to control (corrected by β-actin). Results are means + S.E.M. of at least 3 independent experiments. Statistical significance (P < 0.05, * vs. control, † vs. TRAIL, □ vs. appropriate platinum drug).

Pretreatment of three different human prostate cancer cell lines (DU 145, PC-3, and LNCaP) with subtoxic doses of cisplatin or LA-12 enhanced TRAIL-induced cytotoxicity, as demonstrated by a significant increase in percentage of dead cells (annexin V/propidium iodide assay) and enhanced cleavage of caspase-3 and PARP (Fig 1A–1C) compared to the individual treatments. In all cell lines, the drug combination induced an apparent enhancement of caspase-9 cleavage, indicating the involvement of mitochondrial apoptotic pathway (Fig 1B).

### Cisplatin/LA-12 and TRAIL cooperate to promote stimulation of mitochondrial apoptotic pathway associated with activation of Bid and Bak proteins

**Fig 2:**
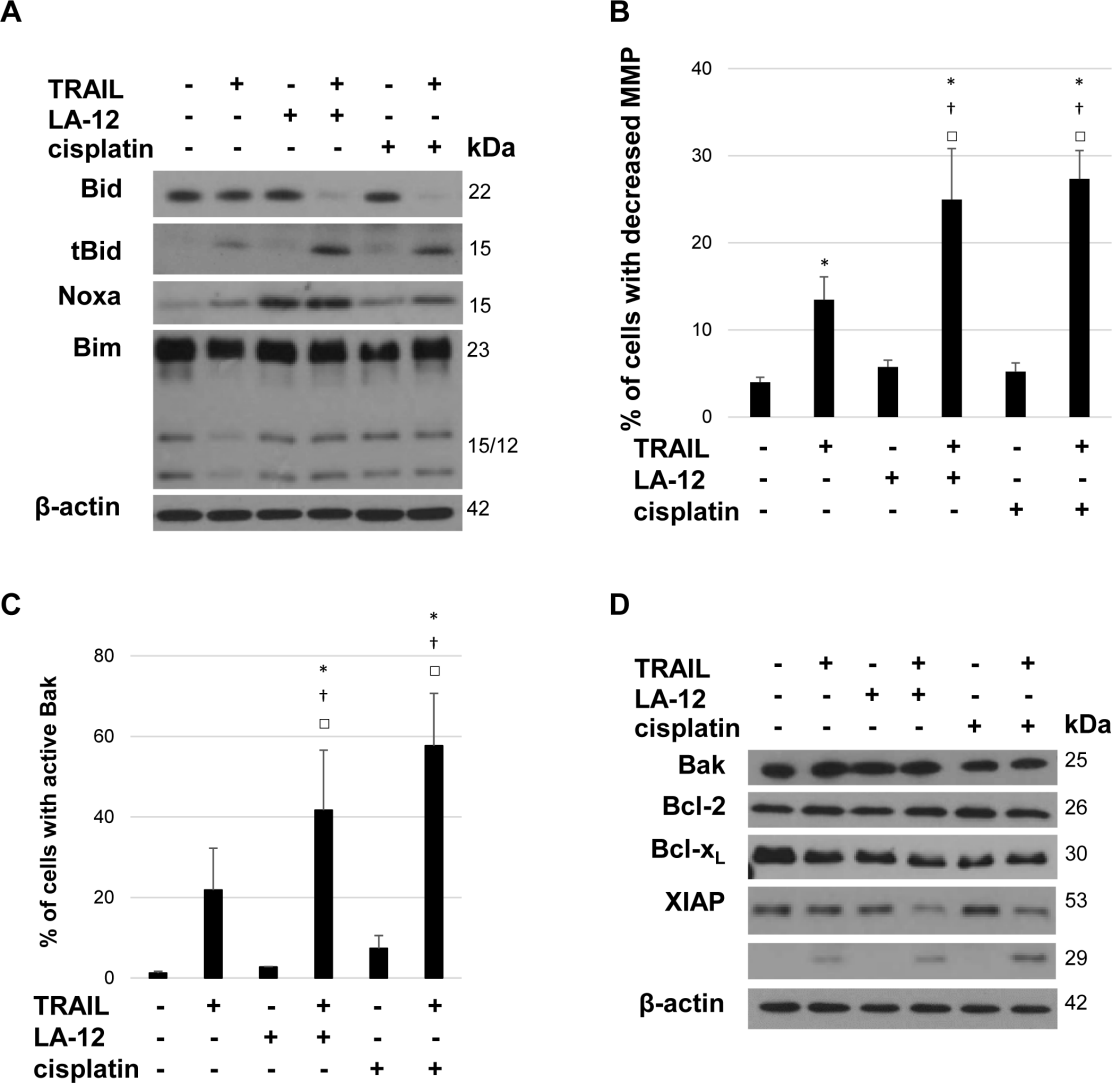
Involvement of mitochondrial apoptotic pathway in LA-12/cisplatin and TRAIL combination-induced DU 145 cell killing. (**A**) The level of Bid/tBid, Noxa, and Bim in DU 145 cells after pretreatment (24 h) with LA-12 (2.5 μM) or cisplatin (5 μM) and subsequent treatment (4 h) with TRAIL (5 ng/ml), detected by Western blotting. (**B, C**) Percentage of DU 145 cells with decreased mitochondrial membrane potential (MMP, TMRE assay, flow cytometry) and active Bak protein (flow cytometry) following treatments specified in A. Results are means + S.E.M. of at least 3 independent experiments. Statistical significance (P < 0.05, * vs. control, † vs. TRAIL, □ vs. appropriate platinum drug). **(D)** The level of Bak, Bcl-2, Bcl-x_L_, and cleavage of XIAP in DU 145 cells treated as in A, detected by Western blotting. The β-actin served as a loading control.

To investigate the role of mitochondria in the cooperative cytotoxic action of cisplatin/LA-12 and TRAIL, we examined several factors related to mitochondrial apoptotic signaling. We observed a significant enhancement of Bid cleavage in all prostate cancer cells lines treated with the combination of cisplatin/LA-12 and TRAIL compared to the agents used alone (Fig 2A and S1 Fig). After treatment with LA-12, the levels of Noxa were upregulated especially in DU 145 and PC-3 cells (Fig 2A and S1 Fig). The treatment of prostate cancer cells with cisplatin/LA-12 and TRAIL did not seem to have any strong impact on the level of Bim (Fig 2A and S1 Fig). Cisplatin/LA-12 and TRAIL combination-induced enhancement of apoptosis was associated with a significant increase in percentage of the cells with decreased MMP (Fig 2B and S2 Fig) and active Bak protein (Fig 2C). The total Bak protein level remained unchanged following the drug treatments in the cell lines studied (Fig 2D and S3 Fig). The treatments with platinum drugs and TRAIL also did not affect the Bax protein level in PC3 and LNCaP cells (S3 Fig). No apparent modulation of the anti-apoptotic Bcl-2 or Bcl-x_L_ protein levels was observed in all prostate cancer cell lines treated with platinum drugs and/or TRAIL (Fig 2D and S3 Fig). We observed a remarkable increase in XIAP protein cleavage in drug combination-treated DU 145 cancer cells compared to the incubation with individual agents (Fig 2D). The decrease in XIAP protein level was also apparent in PC-3 and LNCaP cells treated with the drug combination versus single drugs (S3 Fig). For further more mechanistically-oriented studies focused on the role of mitochondria, we took advantage of the DU 145 cell line that does not express the Bax protein [33], and may serve as a useful tool for further efficient modulation of the mitochondrial apoptotic pathway.

### Apoptosis sensitizing effect of cisplatin/LA-2 and TRAIL combination is suppressed by z-LEHD-fmk but not calpeptin

**Fig 3:**
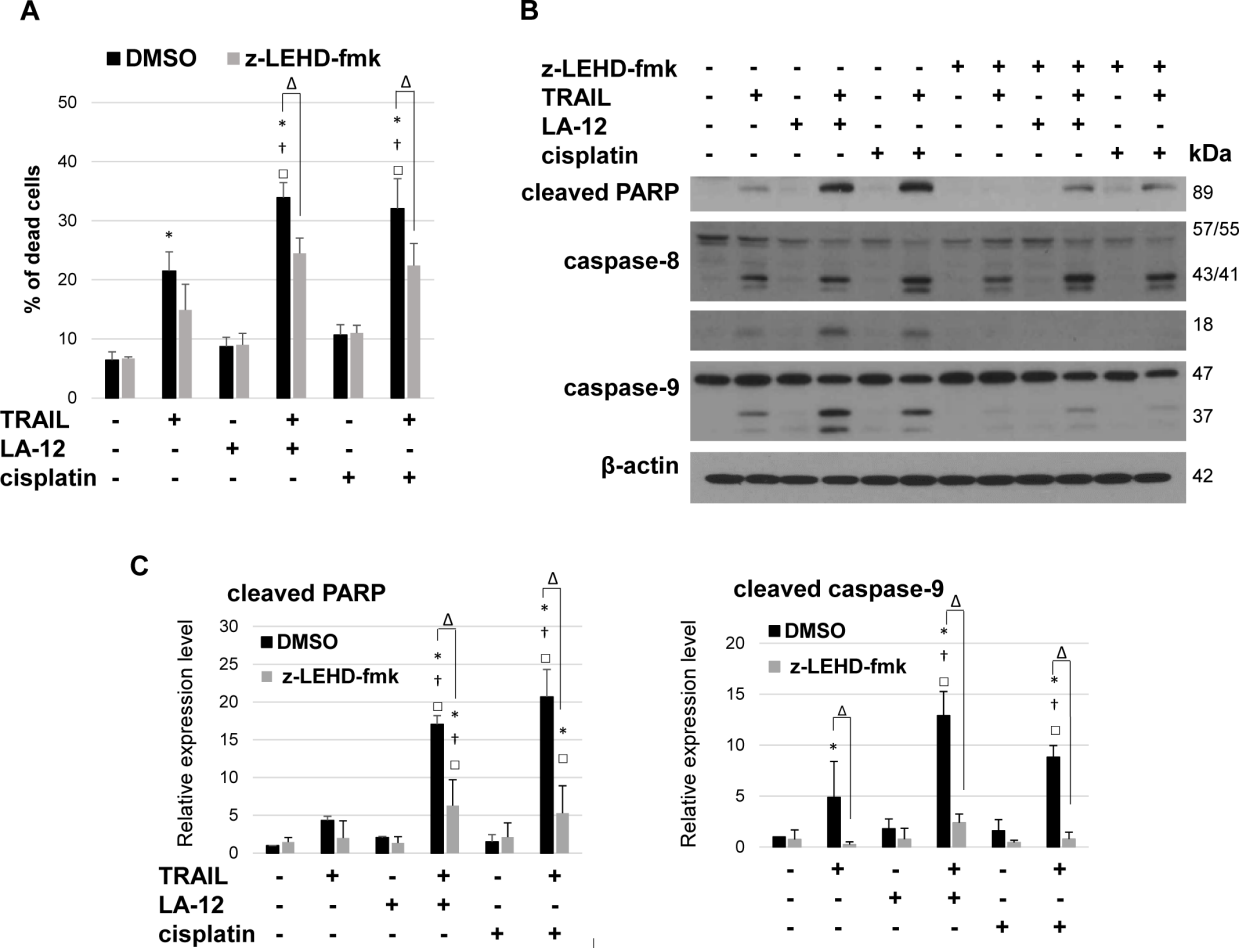
Z-LEHD-fmk mediated suppression of LA-12/cisplatin and TRAIL combination- induced DU 145 cell death. (**A**) Percentage of dead DU 145 cells (flow cytometry, annexin V^+^/PI and annexin V^+^/PI^+^) after pretreatment (1 h) with z-LEHD fmk (20 μM), treatment (24 h) with LA-12 (2.5 μM) or cisplatin (5 μM) and subsequent treatment (4 h) with TRAIL (5 ng/ml). (**B**) Cleavage of PARP, caspase-8, and caspase-9 in DU 145 cells treated as in A, detected by Western blotting. β-actin served as a loading control. Results are representative of at least three independent experiments. **(C)** Densitometry analysis of PARP and caspase-9 cleavage presented in (B). Numbers represent arbitrary units relative to control (corrected by β-actin). Results are means + S.E.M. of 4 independent experiments. Statistical significance (P<0.05, * vs. control, † vs.TRAIL, □ vs. appropriate platinum drug, Δ z-LEHD-fmk- vs. DMSO- pretreated cells).

Pretreatment with caspase-9 inhibitor (z-LEHD-fmk) resulted in pronounced attenuation of cell death in cisplatin/LA-12 and TRAIL combination-treated DU 145 cells, as documented by a significant decrease in their percentage in dead population (Fig 3A), and suppression of PARP cleavage (Fig 3B and 3C). The presence of z-LEHD-fmk also diminished the drug combination-induced cleavage (Fig 3B) and activation (S4A Fig) of caspase-8. The z-LEHD-fmk effectively inhibited caspase-9 cleavage (Fig 3B and 3C) and activity (S4B Fig). In contrast, pretreatment with calpain inhibitor (calpeptin) did not significantly affect the DU 145 cells sensitivity to cisplatin/LA-12 and TRAIL-induced cell death (S5 Fig).

### Silencing of Bak in DU 145 cells significantly suppresses apoptosis but not Bid cleavage induced by cisplatin/LA-12 and TRAIL

**Fig 4:**
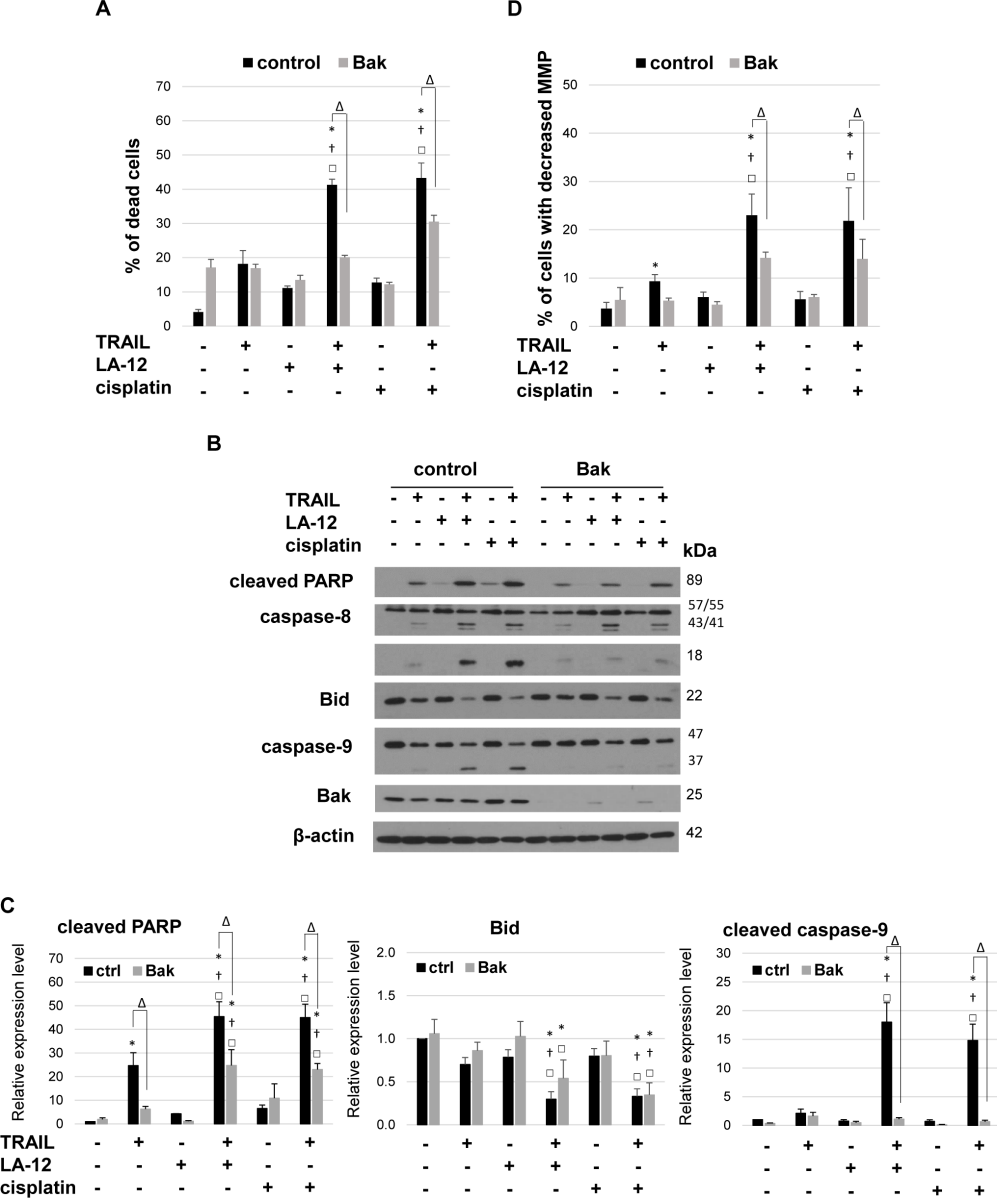
Bak silencing-induced suppression of killing effects of LA-12/cisplatin and TRAIL combination in DU 145 cells. (**A**) Percentage of dead DU 145 cells (annexin V^+^/PI^-^ and annexin V^+^/PI^+^, flow cytometry) transfected with Bak or control siRNA, pretreated (24 h) with LA-12 (2.5 μM) or cisplatin (5 μM) and subsequently treated (4 h) with TRAIL (5 ng/ml). (**B**) Cleavage of PARP, caspase-8, caspase-9, Bid and Bak level in DU 145 cells treated as in (A), detected by Western blotting. The β-actin served as a loading control. (**C**) Densitometry analysis of PARP, caspase-9 and Bid cleavage presented in (B). Numbers represent arbitrary units relative to control (corrected by β-actin). **(D)** Percentage of DU 145 cells with decreased mitochondrial membrane potential (MMP, TMRE assay, flow cytometry) following transfection and treatment specified in A. Results are means + S.E.M. of at least 3 independent experiments. Statistical significance (P < 0.05, * vs. control, † vs. TRAIL, □ vs. appropriate platinum drug, Δ Bak vs. control siRNA-transfected cells).

The specific siRNA-mediated silencing of Bak in Bax-deficient DU 145 cells was responsible for significant suppression of cell death induced by cisplatin/LA-12 and TRAIL combination, as evidenced by results from annexin V/PI assay (Fig 4A) and PARP cleavage (Fig 4B and 4C). The downregulation of Bak also attenuated the drug combination-induced cleavage of caspase-8 (Fig 4B), while it had a minimal impact on Bid cleavage (Fig 4B and 4C). The disruption of mitochondrial apoptotic pathway in the drug combination-treated DU 145 cells with silenced Bak was confirmed by blocked caspase-9 cleavage (Fig 4B and 4C), and impaired loss of MMP (Fig 4D).

### Bid is a critical mediator of apoptosis induced by combination of cisplatin/LA-12 and TRAIL

**Fig 5:**
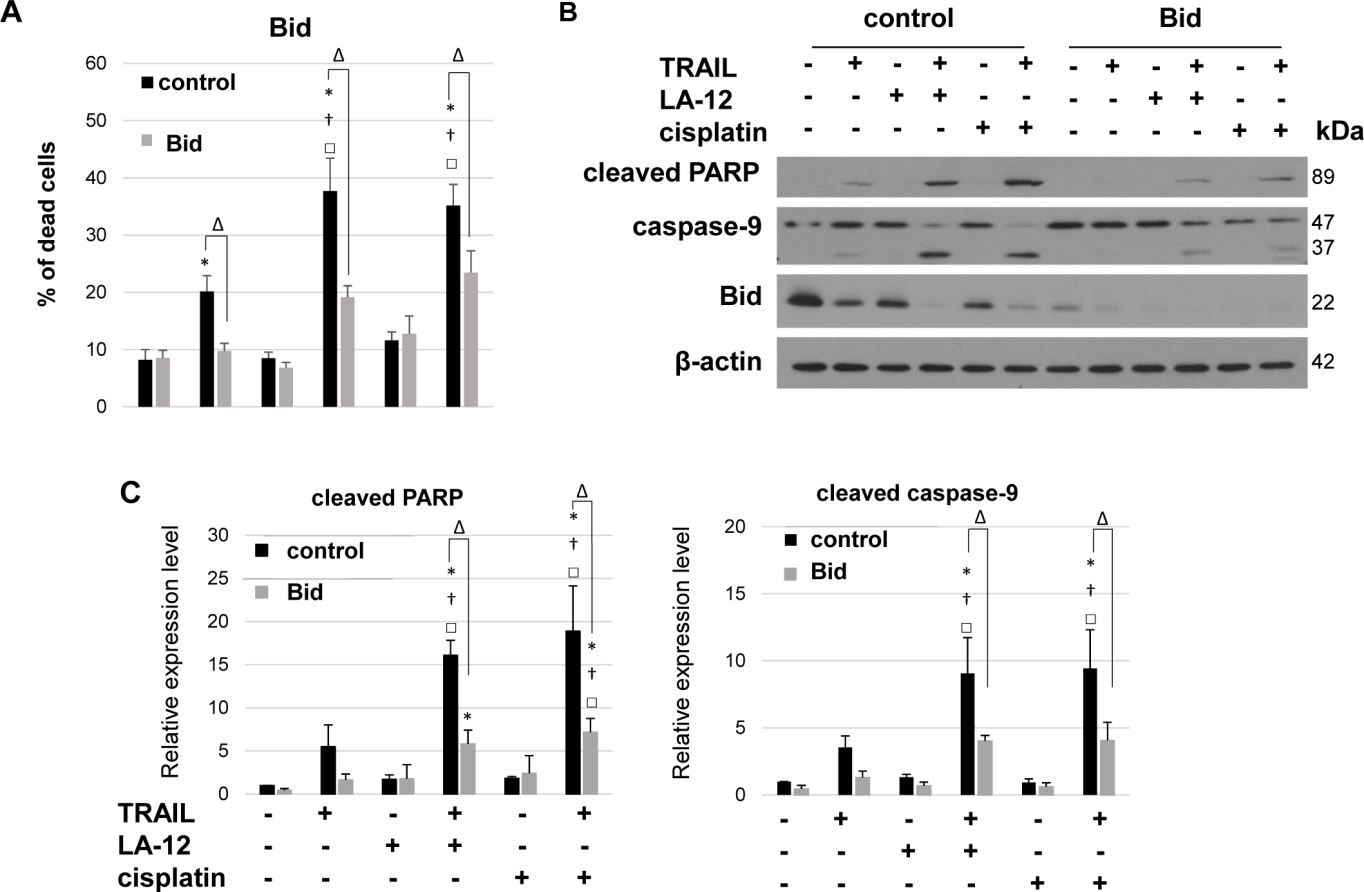
Critical role of Bid in DU 145 cell death induced by combination of LA-12/cisplatin and TRAIL. (**A**) Percentage of dead DU 145 cells (annexin V^+^/PI^-^ and annexin V^+^/PI^+^, flow cytometry) transduced with an empty RNAi vector (control) or vector encoding shRNA targeting Bid, after pretreatment (24 h) with LA-12 (2.5 μM) or cisplatin (5 μM) and subsequent treatment (4 h) with TRAIL (5 ng/ml). (**B**) Cleavage of PARP, caspase-9 and Bid level in DU 145 cells treated as in (A), detected by Western blotting. The β-actin served as a loading control. **(C)** Densitometry analysis of PARP and caspase-9 cleavage presented in (B). Numbers represent arbitrary units relative to control (corrected by β-actin). Results are means + S.E.M. of at least 3 independent experiments. Statistical significance (P < 0.05, * vs. control, † vs. TRAIL, □ vs. appropriate platinum drug, Δ shRNA Bid vs. empty vector-transduced cells).

To examine the functional relevance of Bid in the proapoptotic action of cisplatin/LA-12 and TRAIL, we effectively silenced Bid in DU 145 cells by shRNA. Knockdown of Bid significantly diminished cisplatin/LA-12 and TRAIL-induced increase in cell death (annexin V/PI assay, Fig 5A), cleavage of PARP and caspase-9 (Fig 5B and 5C).

### Caspase-10 is dispensable for enhancement of cisplatin/LA-12 and TRAIL combination-induced apoptosis and Bid cleavage

**Fig 6:**
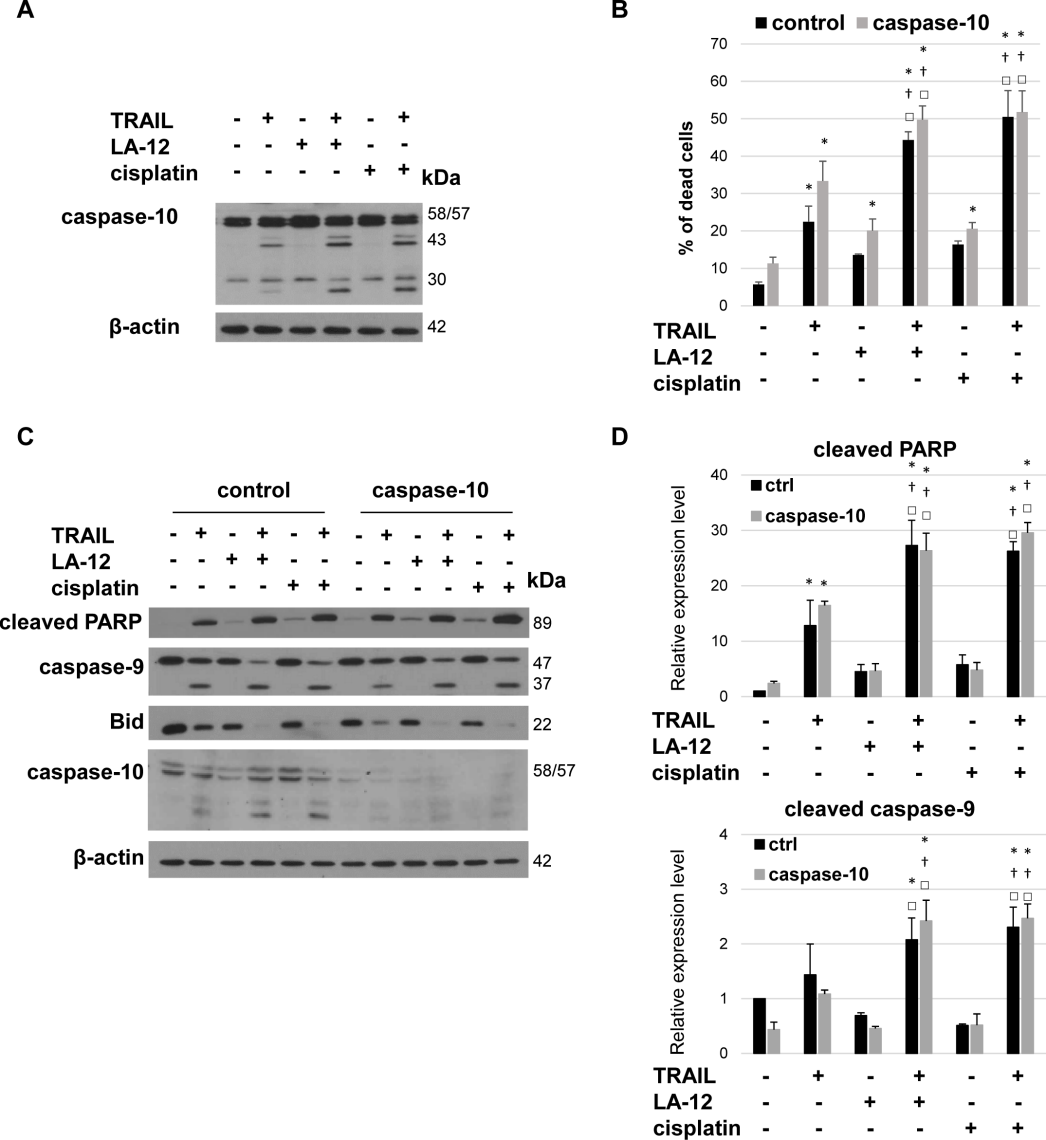
Dispensable role of caspase-10 in LA-12/cisplatin and TRAIL-induced stimulation of DU 145 cell apoptosis and Bid cleavage. (**A**) Cleavage of caspase-10 in DU 145 cells after pretreatment (24 h) with LA-12 (2.5 μM) or cisplatin (5 μM) and subsequent treatment (4 h) with TRAIL (5 ng/ml). (**B**) Percentage of dead DU 145 cells (annexin V^+^/PI^-^ and annexin V^+^/PI^+^, flow cytometry) transfected with caspase-10 or control siRNA, pretreated (24 h) with LA-12 (2.5 μM) or cisplatin (5 μM) and subsequently treated (4 h) with TRAIL (5 ng/ml). (**C**) Cleavage of PARP, caspase-9, and Bid and caspase-10 level in DU 145 cells transfected and treated as in B, detected by Western blotting. The β-actin served as a loading control. **(D)** Densitometry analysis of PARP and caspase-9 cleavage presented in (C). Numbers represent arbitrary units relative to control (corrected by β-actin). Results are means + S.E.M. of at least 3 independent experiments. Statistical significance (P < 0.05, * vs. control, † vs. TRAIL, □ vs. appropriate platinum drug.

We showed that potentiation of cell death in cisplatin/LA-12 and TRAIL-treated prostate cancer cell lines was accompanied by enhanced processing of caspase-10 (Fig 6A and S6 Fig). However, the silencing of this molecule did not interfere with cisplatin/LA-12 and TRAIL combination-induced killing, as no significant changes in overall cell death induction, and cleavage of PARP or caspase-9 were detected in control siRNA versus caspase-10 siRNA-transfected DU 145 cells (Fig 6B–6D). Caspase-10 silencing also did not affect the Bid cleavage in the cells treated with combination of TRAIL and cisplatin/LA-12 (Fig 6C).

### LA-12 sensitizes primary human prostate cancer cells to TRAIL-induced apoptosis

**Fig 7:**
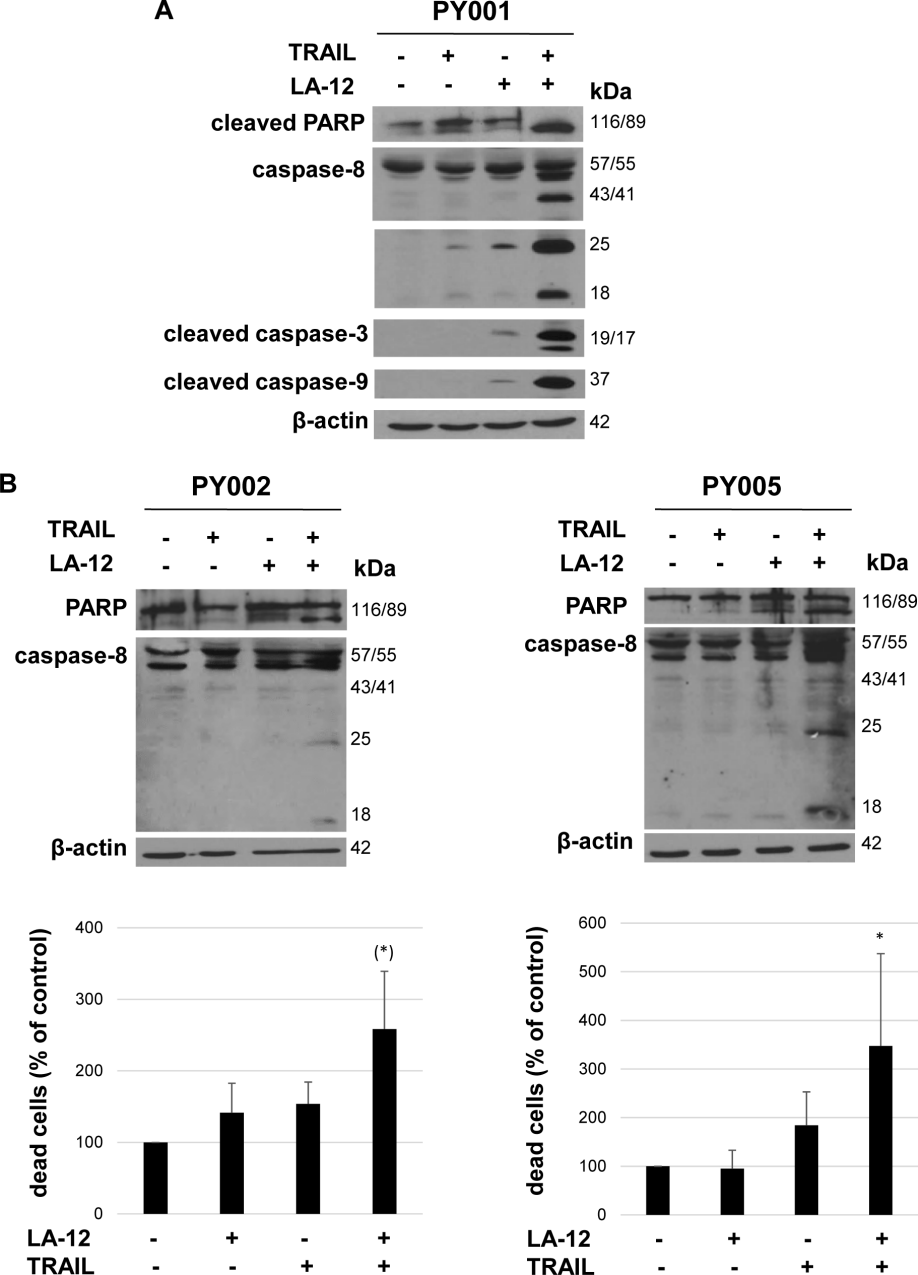
LA-12-mediated sensitization of human primary prostate cancer cells to TRAIL-induced apoptosis. (**A**) Cleavage of PARP, caspase-8, -3, -9 in PY001 prostate cancer cells pretreated (24 h) with LA-12 (5 μM) and subsequently treated (24 h) with TRAIL (200 ng/ml), detected by Western blotting. The β-actin served as a loading control. Shorter (full length) or longer (cleaved fragments) exposition times are presented for caspase-8. (**B**) Cleavage of PARP and caspase-8 (Western blotting), and dead (annexin V^+^/PI^-^ and annexin V^+^/PI^+^, flow cytometry) PY002 or PY005 prostate cancer cells (expressed as a percentage of control) treated as in A. Results are means + S.E.M. or representatives of 3 independent experiments. Statistical analysis: (*) vs. control, P = 0.126 for PY002; * vs. control, P = 0.047 for PY005.

The combination of LA-12 with TRAIL enhanced the cytotoxic response also in primary human prostate cancer cells, which was documented by an enhanced cleavage of PARP and caspase-8 (Fig 7A and 7B), and caspase-3, -9 (Fig 7A) or percentage of dead cells in annexin V/PI assay (Fig 7B) in drug combination versus individually treated samples of three patients marked as PY001, PY002 and PY005. These cells were apparently resistant to the cytotoxic/cytostatic effects of TRAIL alone used up to dose of 1 μg/ml, as documented by CyQuant Cell Proliferation Assay Kit (S7 Fig).

## Discussion

In order to propose novel strategies for prostate cancer cell sensitization to TRAIL-induced killing, we examined the cooperative cytotoxic effects of platinum drugs and this cytokine, and related molecular mechanisms. Our previous work showed the ability of cisplatin or LA-12 to effectively enhance TRAIL-induced apoptosis in human PC3 prostate cancer cells, which was associated with modulations of some upstream events in the extrinsic apoptotic pathway such as re-localization of DR4/5 into lipid rafts or TRAIL internalization [25]. The present work was aimed to investigate the involvement of mitochondria in platinum-based drug-mediated stimulation of TRAIL-induced apoptosis in prostate cancer cells. Here we demonstrate that cisplatin/LA-12 and TRAIL combination-induced cytotoxicity was associated with profound stimulation of the mitochondrial apoptotic pathway in several human prostate cancer cell lines, as evidenced by the enhanced cleavage of Bid, activation of Bak, drop of mitochondrial membrane potential, and caspase-9 activation. Although some other studies also suggested an involvement of the intrinsic apoptotic pathway in the cisplatin-mediated sensitization to TRAIL- or lexatumumab-induced apoptosis in DU 145 or PC-3 cells [26, 27], the molecular mechanisms behind still remain to be understood. Moreover, till present, no relevant information has been published concerning the involvement of these organelles in the cooperative cytotoxic action of LA-12 and TRAIL in prostate cancer cells.

We showed that the killing effect of cisplatin/LA-12 and TRAIL in DU 145 cells was suppressed in the presence of z-LEHD-fmk, which indicated the functional role of mitochondria and caspase-9 in apoptotic machinery triggered by the drug combination. However, being aware of the fact that under some circumstances, z-LEHD-fmk might also inhibit other targets in addition to caspase-9 [34], we enlarged our observations using other cellular models with specifically blocked mitochondrial functions. We took advantage of the DU 145 cell line that carries a monoallelic Bax frameshift mutation and a second silent Bax allele, thus being completely devoid of Bax protein expression [33]. The Bax and Bak are known as the two main effectors of the Bcl-2 family at the level of mitochondria, crucial for further propagation of apoptotic signal downstream of these organelles [35]. Therefore, in Bax-deficient DU 145 cells, the silencing of Bak may serve as a useful approach to block the mitochondrial apoptotic pathway prior to the drug treatments, and further study the consequences. We showed that siRNA-mediated silencing of Bak in DU 145 cells significantly suppressed caspase-9 activation and apoptosis induced by the drug combination. Similar apoptosis-suppressing effects were also obtained in the drug combination-treated DU 145 cells with silenced Bid, a crucial BH3-only protein connecting proximal DR signals to the mitochondrial apoptotic machinery via its interaction with Bcl-2 family members such as Bax or Bak [6, 7]. Thus, using three different approaches we unequivocally demonstrated the indispensable role of mitochondria in the efficient realization of cytotoxic potential of the combination of cisplatin/LA-12 and TRAIL in prostate cancer cells.

We further showed that pretreatment with z-LEHD-fmk or siRNA-mediated silencing of Bak resulted in significant suppression of the enhanced caspase-8 cleavage observed in cisplatin/LA-12 and TRAIL combination-treated DU 145 cells. It means that activation of caspase-8 in the drug combination-treated cells was mediated not only via its DR-DISC-dependent processing, but also due to Bak-dependent mitochondrial pathway and resulting caspase-9 activation. Previous studies also demonstrated that caspase-8 activation induced by various apoptotic stimuli including TRAIL and cisplatin required positive mitochondrial feedback amplification loop [27, 30, 36–38]. In this system, caspase-9, -3 and -6 were implicated in the caspase-8 activation downstream of mitochondria [39–41]. Based on the data obtained by us and the above-mentioned studies, we suggest that stimulation of mitochondrial pathway-derived caspase-9 and resulting effector caspase activity is required for the efficient caspase-8 activation in the cisplatin/LA-12 and TRAIL combination-treated prostate cancer cells. We also showed that in contrast to caspase-8 processing, the cleavage of Bid was not attenuated in the drug combination-treated DU 145 cells with silenced Bak. This indicates that the secondarily activated caspase-8 did not significantly contribute to Bid cleavage in the drug combination-treated DU 145 cells. Instead, we suggest the existence of complex feedback mechanism where secondarily activated caspase-8 can further cleave the effector caspases, additionally amplifying the caspase cascade in the prostate cancer cells treated with cisplatin/LA-12 and TRAIL combination.

We assumed that the specific modulations of the ratio between proapoptotic and antiapoptotic Bcl-2 family proteins in cancer cells treated with cisplatin/LA-12 and TRAIL may help to render mitochondria more susceptible to apoptotic signal transmitted from DRs via tBid. In addition to Bid, which we identified as a crucial mediator of cisplatin/LA-12- and TRAIL-induced apoptosis in prostate cancer cells, we examined the potential changes in the level of other BH3 only Bcl-2 family members. In accordance with our previous work in colon cancer models [30], we also observed that LA-12 acts as an inducer of Noxa in some prostate cancer cells, while the impact of cisplatin on this protein level was less evident. Cisplatin-mediated upregulation of Noxa was functionally important for the killing activity of this drug in other cancer cells [42], while it was not essential for cisplatin-mediated colon cancer cell sensitization to TRAIL-induced apoptosis [30]. Previously, we also demonstrated the ability of LA-12 to upregulate the BH3 only Bim_L_ level in colon cancer cells [30], which was not obvious in the present study with prostate models, further excluding its major role in the drug-mediated cytotoxicity. In addition, we could not detect any significant changes in the amount of antiapoptotic Bcl-2 family proteins Bcl-2 and Bcl-xL in the cisplatin/LA-12 and TRAIL combination-treated prostate cancer cells. Similar results were also reported in human TE12 esophageal or HCT116 colon cancer cells treated with the combination of TRAIL with cisplatin or LA-12 [30, 38]. In contrast, reduction of the level of these proteins was suggested to contribute to cisplatin-mediated enhancement of TRAIL-induced apoptosis in human CRL2335 and MDA-MB-468 triple negative breast or SKOV3 ovarian cancer cells [43, 44]. We concluded that the role of Bcl-2 or Bcl-xL in modulation of prostate cancer cell sensitivity to combined cisplatin/LA-12 and TRAIL treatment may be cell type-specific, but not crucial in prostate cancer cell lines used within our study.

In type II cells that rely on mitochondria for further amplification of DR-mediated apoptotic signal, regulation of XIAP represents an important control point in apoptosis execution [45]. XIAP is the most potent and most studied inhibitor of caspases among human cIAP family, and promising therapeutic target in cancer treatment [46]. Abrogation of its expression by antisense phosphorodiamidate morpholino oligomer was essential for DU 145 prostate cancer cell sensitization to the cytotoxic effects of the individually applied cisplatin or TRAIL [47]. The ability of cisplatin to induce apoptosis through the inhibition of XIAP expression has also been reported in LNCaP cells [48]. Interestingly, caspase-3-dependent cleavage of XIAP, followed by its proteasomal degradation, has been shown to accompany cisplatin-mediated sensitization of melanoma cells to TRAIL-induced apoptosis [49]. In the present study, the enhancement of apoptosis induced by cisplatin/LA-12 and TRAIL was associated with potentiation of XIAP cleavage that coincided with caspase activity. We suggest that the loss of XIAP we observed in prostate cancer cells treated with the drug combination might contribute to their increased apoptotic response compared to the individual drug applications.

An apparent enhancement of caspase-10 cleavage in cisplatin/LA-12 and TRAIL-treated prostate cancer cells prompted us to investigate in more detail its possible involvement in the cooperative cytotoxic action of the drugs. While the function of caspase-8 in TRAIL-induced signaling and Bid cleavage is well established, the role of caspase-10 is more controversial and less understood. Whereas some studies indicated that caspase-10 is crucial for TRAIL signaling [50–52], other reports suggest that it is not important [53, 54]. The ability of caspase-10 to trigger Bid cleavage and caspase cascade activation in FasL-induced apoptosis has also been reported [55]. Interestingly, caspase-10 has recently been shown to negatively regulate caspase-8-mediated cell death [56]. In present study, we newly showed that caspase-10 was dispensable for realization of the cytotoxic potential of the cisplatin/LA-12 and TRAIL combination in prostate cancer cells, and also did not significantly affect the drug-induced Bid cleavage. Furthermore, calpain-mediated Bid cleavage has been shown as functionally important in cisplatin-induced apoptosis of human melanoma cells, which was blocked by calpain inhibitors [57]. We demonstrated that cisplatin/LA-12 and TRAIL combination-induced enhancement of cell death was not affected by calpeptin, further excluding the possible importance of calpain-mediated Bid cleavage in drug combination treated prostate cancer cells.

A very frequent resistance to TRAIL-induced apoptosis was previously reported in a large cohort of patient prostate cancer tissue samples [10], and our observations also support these findings. Although TRAIL monotherapy seems to be inefficient for treatment of this type of cancer, our results highlight a possible applicability of platinum-based complexes in sensitization of prostate cancer cells to TRAIL-induced apoptosis. Importantly, we newly showed that cisplatin/LA-12 and TRAIL cooperate to induce cell death in primary prostate cancer cell cultures isolated from patient tumor biopsies. This drug combination-induced stimulation of the cytotoxic response was associated with activation of caspases including caspase-9, indicating the involvement of mitochondrial apoptotic pathway. As cisplatin is commonly used drug in cancer treatment, and both TRAIL and LA-12 have individually entered clinical trials, we suggest that the drug combinations examined in our study could be considered as an interesting approach for future treatment strategies.

**Fig 8:**
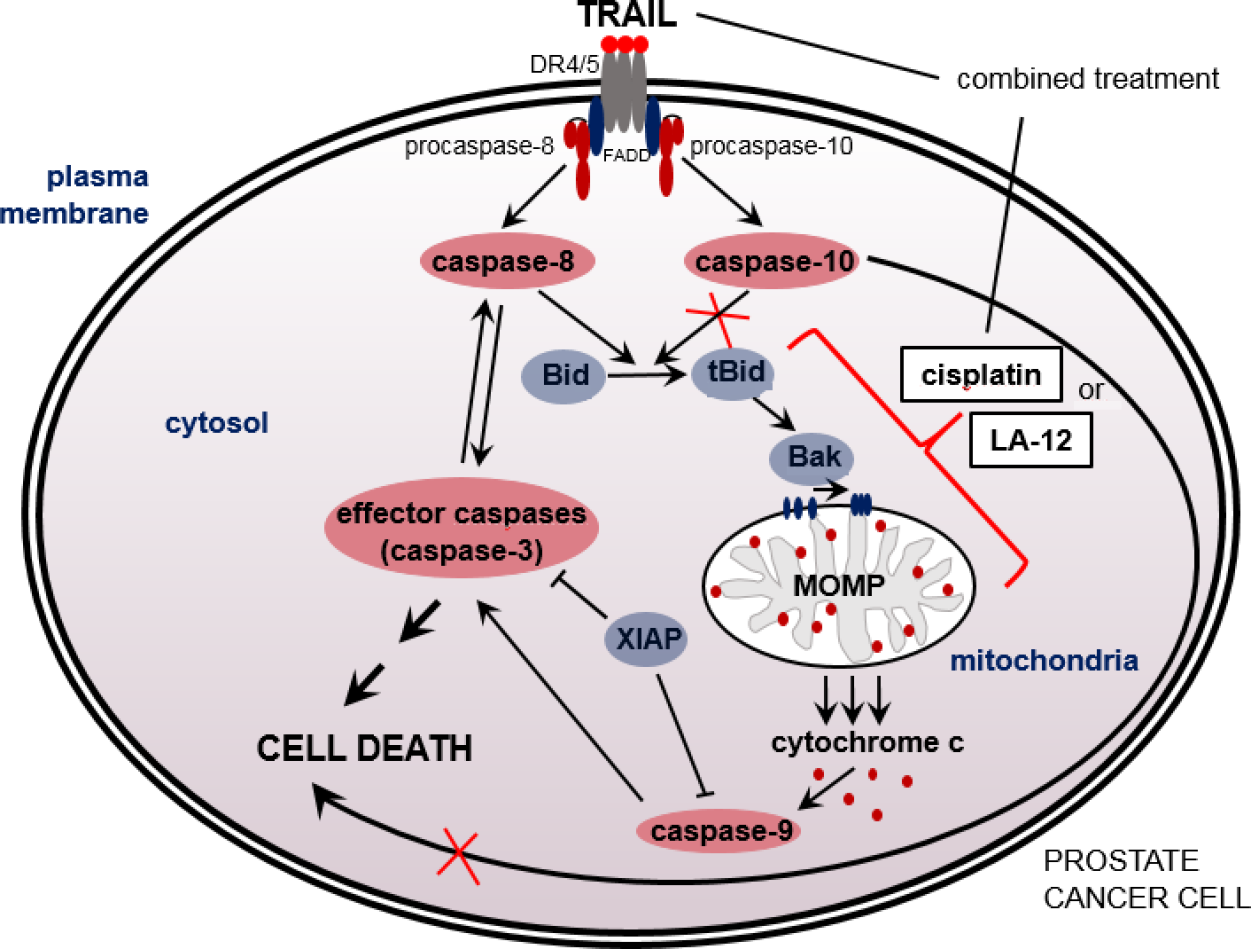
Cisplatin/LA-12-mediated enhancement of TRAIL-induced apoptosis in prostate cancer cells–main molecular mechanisms involved. Cisplatin or LA-12 enhance the sensitivity of human prostate cancer cells to TRAIL- induced cell death via an engagement of mitochondrial apoptotic pathway. This was accompanied by augmented Bid cleavage, Bak activation, loss of mitochondrial membrane potential, activation of caspase-8, -10, -9, and -3, and XIAP cleavage. The caspase-10 was dispensable for enhancement of cisplatin/LA-12 and TRAIL combination-induced cell death and stimulation of Bid cleavage.

In summary, we showed that pretreatment of human prostate cancer cells with cisplatin or LA-12 resulted in significant potentiation of TRAIL-induced cell death via engagement of mitochondrial apoptotic pathway, associated with pronounced Bid cleavage, Bak activation, loss of MMP and caspase activation. RNAi-mediated silencing of Bid or Bak in Bax-deficient DU 145 cells suppressed drug combination-induced cytotoxicity, further underscoring the importance of mitochondrial signaling. The cisplatin/LA-12 and TRAIL combination-induced stimulation of Bid cleavage did not seem to essentially require caspase-10 or Bak. We also newly showed that caspase-10 was dispensable for platinum drug-mediated stimulation of TRAIL-induced apoptosis in prostate cancer cells (Fig 8). Compared to cisplatin, lower doses of LA-12 were required for similar sensitizing effects on TRAIL-induced prostate cancer cell apoptosis. Importantly, an enhancement of TRAIL-induced apoptosis mediated by LA-12 was for the first time demonstrated in cancer cells derived from prostate tumor specimens obtained from human patients. Our results indicate that anticancer strategies employing TRAIL combined with platinum-based drugs could contribute to more effective elimination of prostate cancer cells compared to the individual action of the drugs, which warrants further investigation.

## Supporting information

S1 Fig Modulation of selected BH3-only protein levels in PC-3 and LNCaP cells treated with LA-12/cisplatin and TRAIL. (PDF)

S2 Fig Changes in mitochondrial membrane potential (MMP) in PC-3 and LNCaP cells treated with LA-12/cisplatin and TRAIL. (PDF)

S3 Fig Levels of selected pro- and anti-apoptotic proteins in PC-3 and LNCaP cells treated with LA-12/cisplatin and TRAIL. (PDF)

S4 Fig Z-LEHD-fmk mediated inhibition of caspase-8 activity in LA-12/cisplatin and TRAIL combination-induced DU 145 cells. (PDF)

S5 Fig Pretreatment with calpeptin did not affect apoptosis induced by combination of LA-12/cisplatin and TRAIL. (PDF)

S6 Fig Caspase-10 cleavage induced by LA-12/cisplatin and TRAIL in PC-3 and LNCaP cells. (PDF)

S7 Fig Primary human prostate cancer cells were resistant to cytotoxic/cytostatic effects of TRAIL. (PDF)

S8 Fig Original blots with markers for results presented in Figs 1–7. (PDF)

S9 Fig Original blots with markers for results presented in Supplementary figures. (PDF)

S10 Fig Supplementary material and methods. (PDF)
