# Supplementary material for: Cisplatin or LA-12 enhance killing effects of TRAIL in prostate cancer cells through Bid-dependent stimulation of mitochondrial apoptotic pathway but not caspase-10
Source: PLoS One. 2017 Nov 28;12(11):e0188584. doi: 10.1371/journal.pone.0188584 (PMC5705153; doi:10.1371/journal.pone.0188584)
Supplement: S2 Fig — (PDF) [file pone.0188584.s002.pdf]

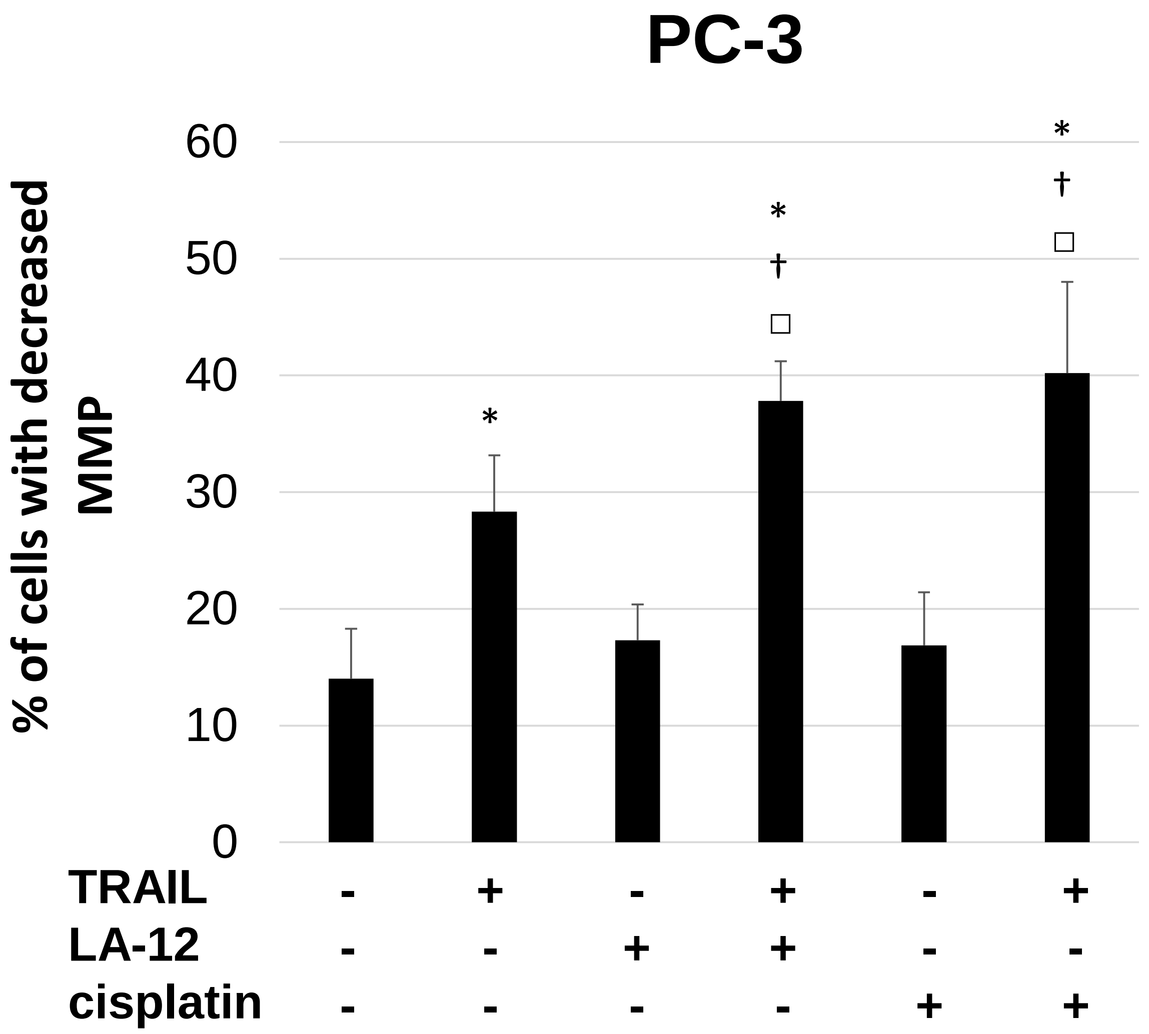

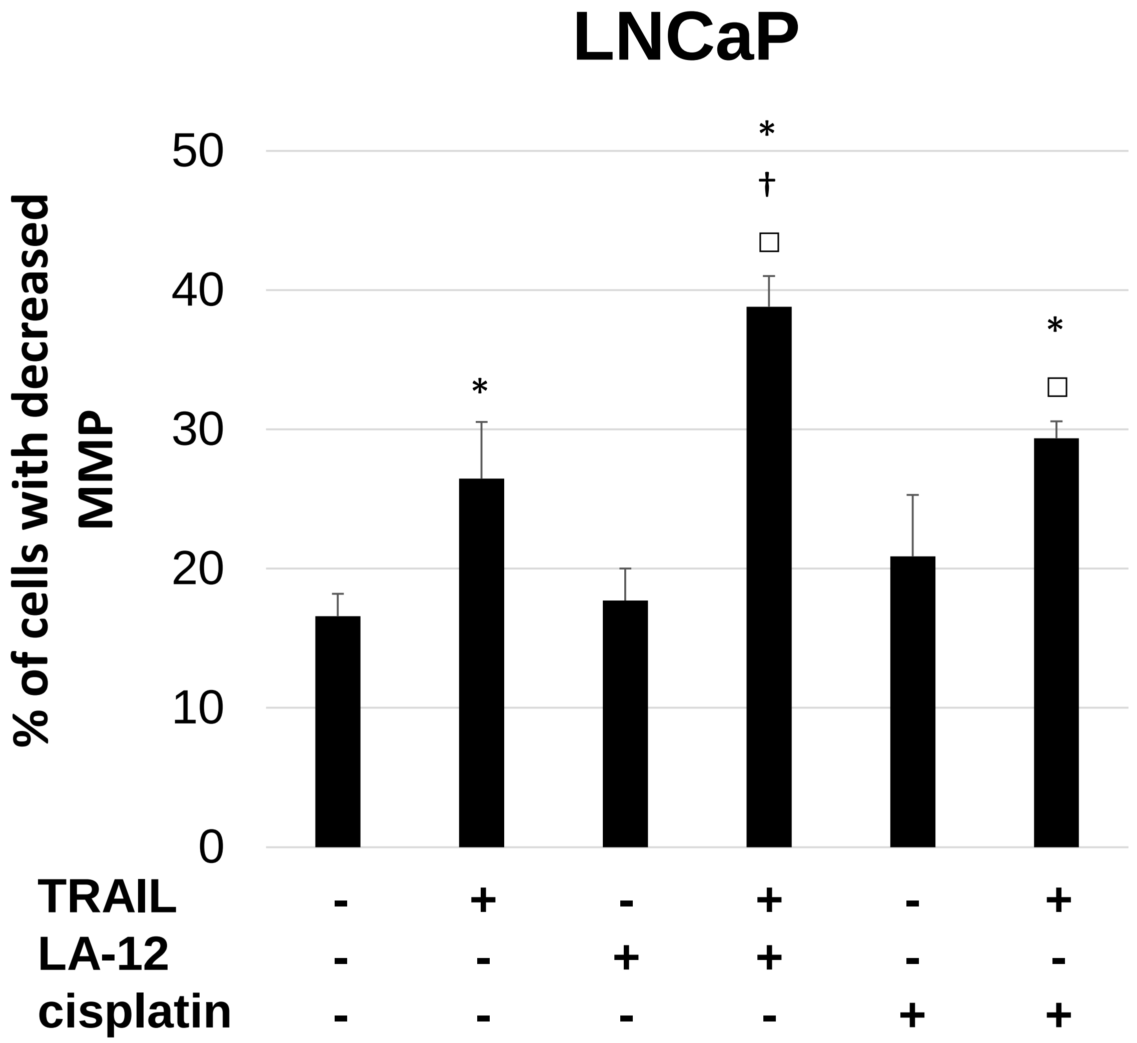

S2 Changes in mitochondrial membrane potential (MMP) in PC-3 and LNCaP cells treated with LA-12/cisplatin and TRAIL. Percentage of PC-3 and LNCaP cells with decreased MMP (TMRE assay, flow cytometry) following pretreatment (24 h) with LA-12 (0.5/2.5 μM) or cisplatin (10/5 μM) and treatment (4 h) with TRAIL (5/20 ng/ml), respectively. Results are means + S.E.M. of 3 independent experiments. Statistical significance ($P < 0.05$, * vs. control, † vs. TRAIL, □ vs. appropriate platinum drug).
