# Supplementary material for: Cisplatin or LA-12 enhance killing effects of TRAIL in prostate cancer cells through Bid-dependent stimulation of mitochondrial apoptotic pathway but not caspase-10
Source: PLoS One. 2017 Nov 28;12(11):e0188584. doi: 10.1371/journal.pone.0188584 (PMC5705153; doi:10.1371/journal.pone.0188584)
Supplement: S3 Fig — (PDF) [file pone.0188584.s003.pdf]

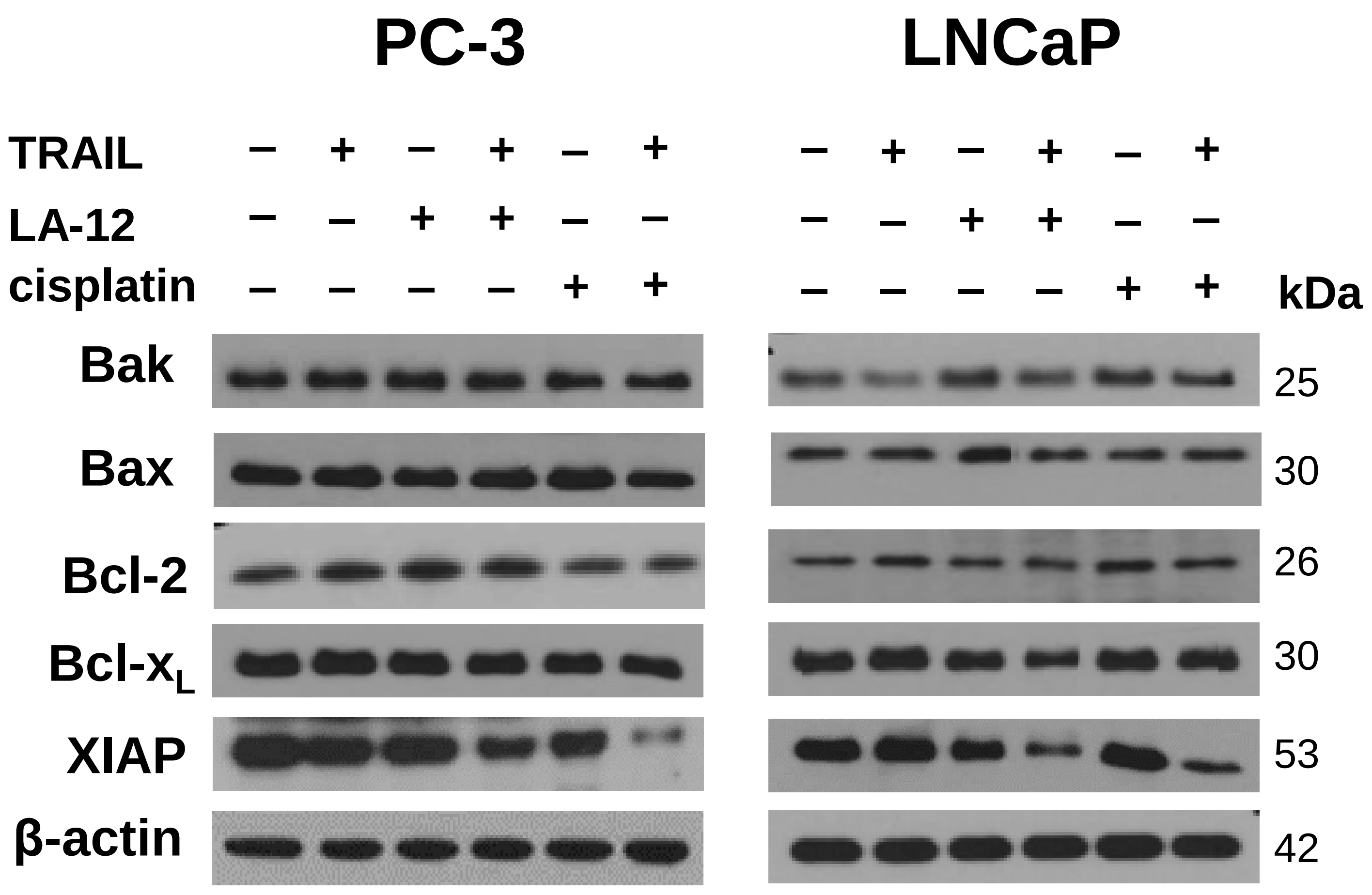

S3 Levels of selected pro- and anti-apoptotic proteins in PC-3 and LNCaP cells treated with LA-12/cisplatin and TRAIL. The level of Bak, Bax, Bcl-2, Bcl-xL and XIAP in PC-3 and LNCaP cells pretreated (24 h) with LA-12 (0.5/2.5 μM) or cisplatin (10/5 μM) and treated (4 h) with TRAIL (5/20 ng/ml), respectively, detected by Western blotting. β-actin served as a loading control. Results are representative of at least three independent experiments.
