# Supplementary material for: Cisplatin or LA-12 enhance killing effects of TRAIL in prostate cancer cells through Bid-dependent stimulation of mitochondrial apoptotic pathway but not caspase-10
Source: PLoS One. 2017 Nov 28;12(11):e0188584. doi: 10.1371/journal.pone.0188584 (PMC5705153; doi:10.1371/journal.pone.0188584)
Supplement: S4 Fig — (PDF) [file pone.0188584.s004.pdf]

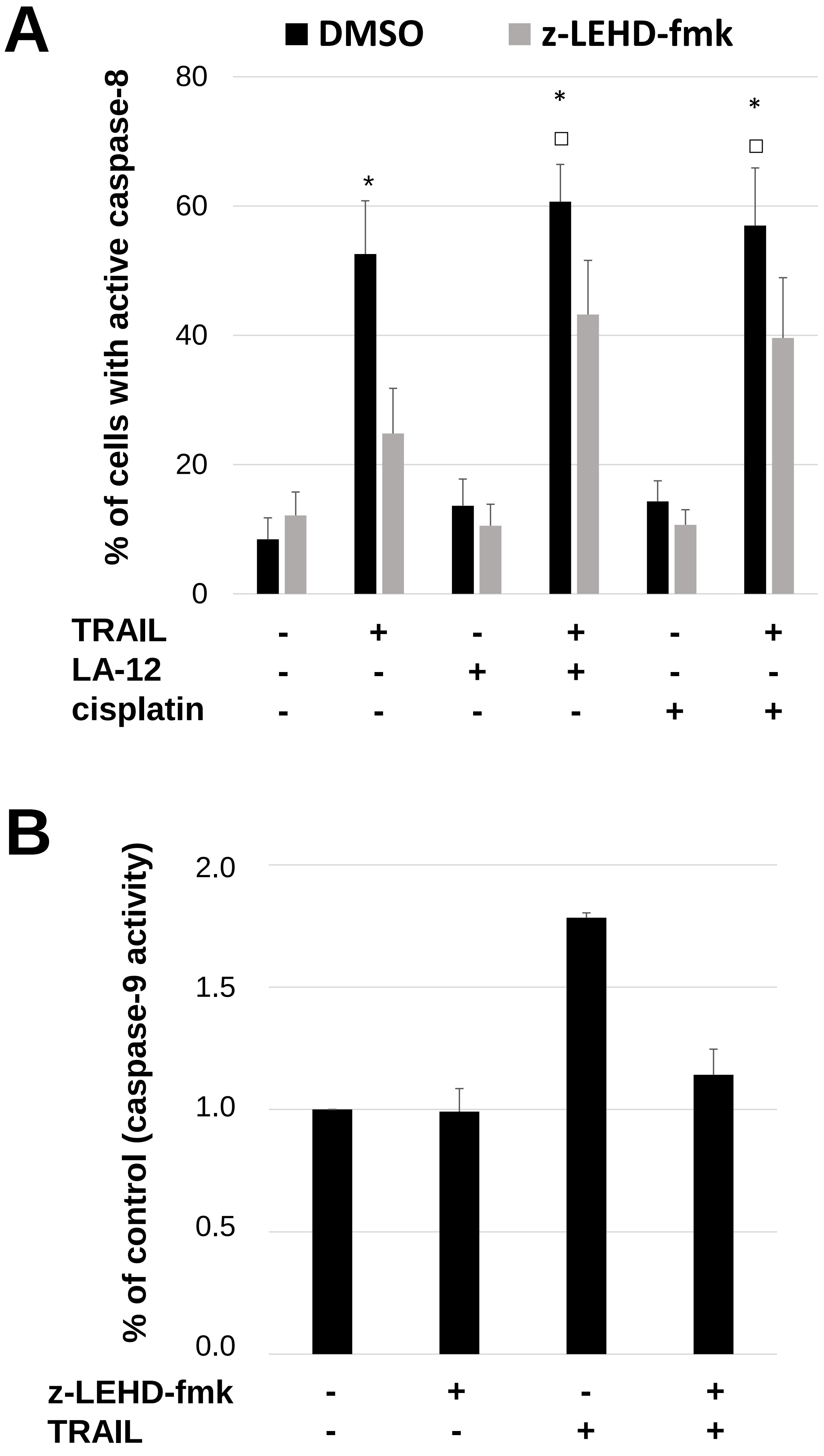

S4 Z-LEHD-fmk mediated inhibition of caspase-8 activity in LA-12/cisplatin and TRAIL combination-induced DU 145 cells. (**A**) Percentage of DU 145 cells with active caspase-8 (FLICA, flow cytometry) after pretreatment (1 h) with z-LEHD-fmk (20 μM), treatment (24 h) with LA-12 (2.5 μM) or cisplatin (5 μM) and subsequent treatment (4 h) with TRAIL (5 ng/ml). Results are means + S.E.M. of 3 independent experiments. Statistical significance ($P < 0.05$, * vs. control, □ vs. appropriate platinum drug). (**B**) Activity of caspase-9 (specific fluorogenic substrate) in HCT116 cells pretreated (1 h) with z-LEHD-fmk (20 μM) and then treated (24 h) with TRAIL (50 ng/ml). Results are means + S.E.M. of 2 independent experiments.
