# Supplementary material for: Cisplatin or LA-12 enhance killing effects of TRAIL in prostate cancer cells through Bid-dependent stimulation of mitochondrial apoptotic pathway but not caspase-10
Source: PLoS One. 2017 Nov 28;12(11):e0188584. doi: 10.1371/journal.pone.0188584 (PMC5705153; doi:10.1371/journal.pone.0188584)
Supplement: S5 Fig — (PDF) [file pone.0188584.s005.pdf]

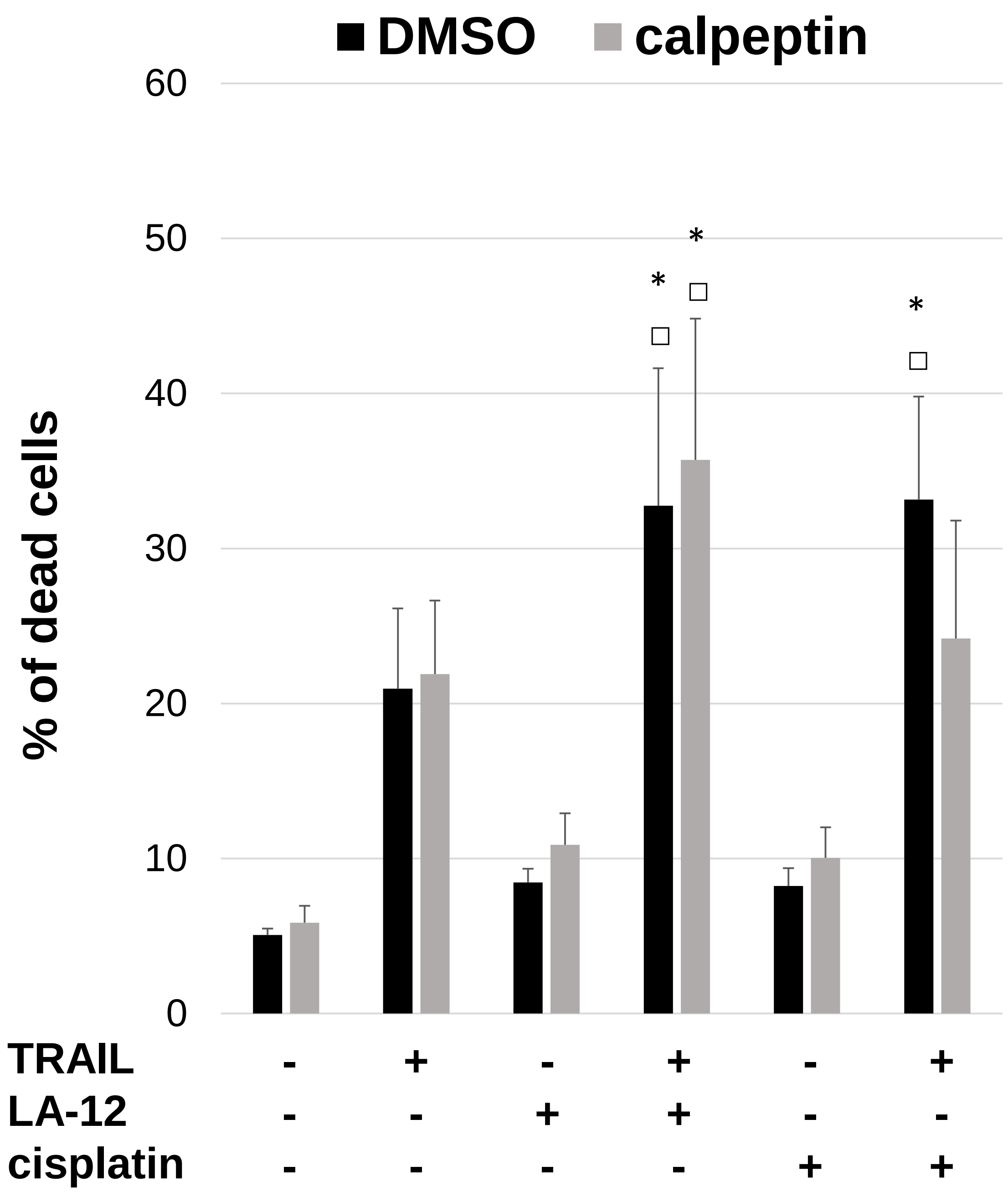

S5 Pretreatment with calpeptin did not affect apoptosis induced by combination of LA-12/cisplatin and TRAIL. Percentage of dead DU 145 cells (flow cytometry, annexin V^+/PI^- and annexin V^+/PI^+) after pretreatment (1 h) with calpeptin (10 μM), treatment (24 h) with LA-12 (2.5 μM) or cisplatin (5 μM) and subsequent treatment (4 h) with TRAIL (5 ng/ml). Results are means + S.E.M. of 3 independent experiments. Statistical significance ($P < 0.05$, * vs. control, □ vs. appropriate platinum drug).
