# Supplementary material for: Cisplatin or LA-12 enhance killing effects of TRAIL in prostate cancer cells through Bid-dependent stimulation of mitochondrial apoptotic pathway but not caspase-10
Source: PLoS One. 2017 Nov 28;12(11):e0188584. doi: 10.1371/journal.pone.0188584 (PMC5705153; doi:10.1371/journal.pone.0188584)
Supplement: S7 Fig — (PDF) [file pone.0188584.s007.pdf]

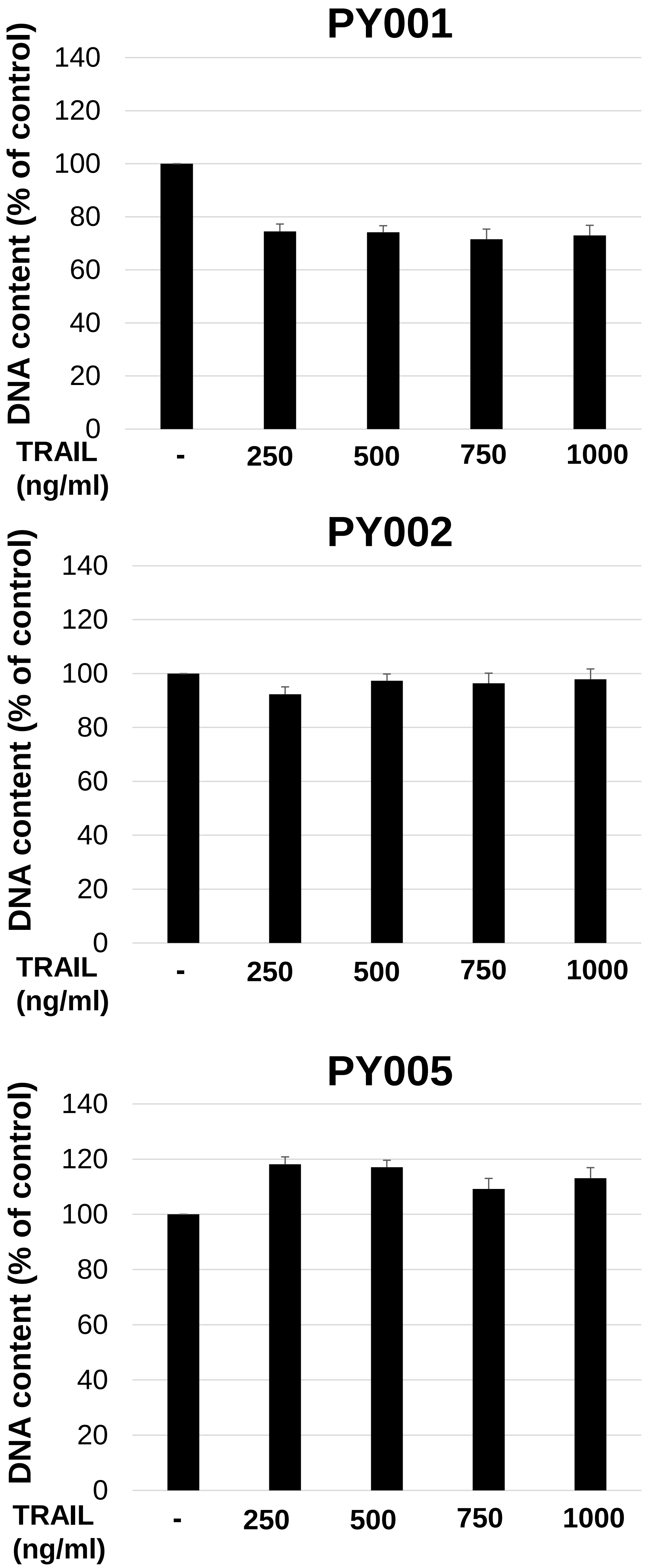

S7 Primary human prostate cancer cells were resistant to cytotoxic/cytostatic effects of TRAIL. Total DNA content (percentage of vehicle-treated control) in patient sample PY001, PY002 and PY005 after treatment (24 h) with TRAIL (250; 500; 750; 1000 ng/ml), analyzed by CyQUANT assay. Results are means + S.E.M. of 3 independent experiments.
