# Supplementary figures and images for: Cisplatin or LA-12 enhance killing effects of TRAIL in prostate cancer cells through Bid-dependent stimulation of mitochondrial apoptotic pathway but not caspase-10
Source: PLoS One. 2017 Nov 28;12(11):e0188584. doi: 10.1371/journal.pone.0188584 (PMC5705153; doi:10.1371/journal.pone.0188584)

S9 Fig Original blots with markers for results presented in Supplementary figures.

S1

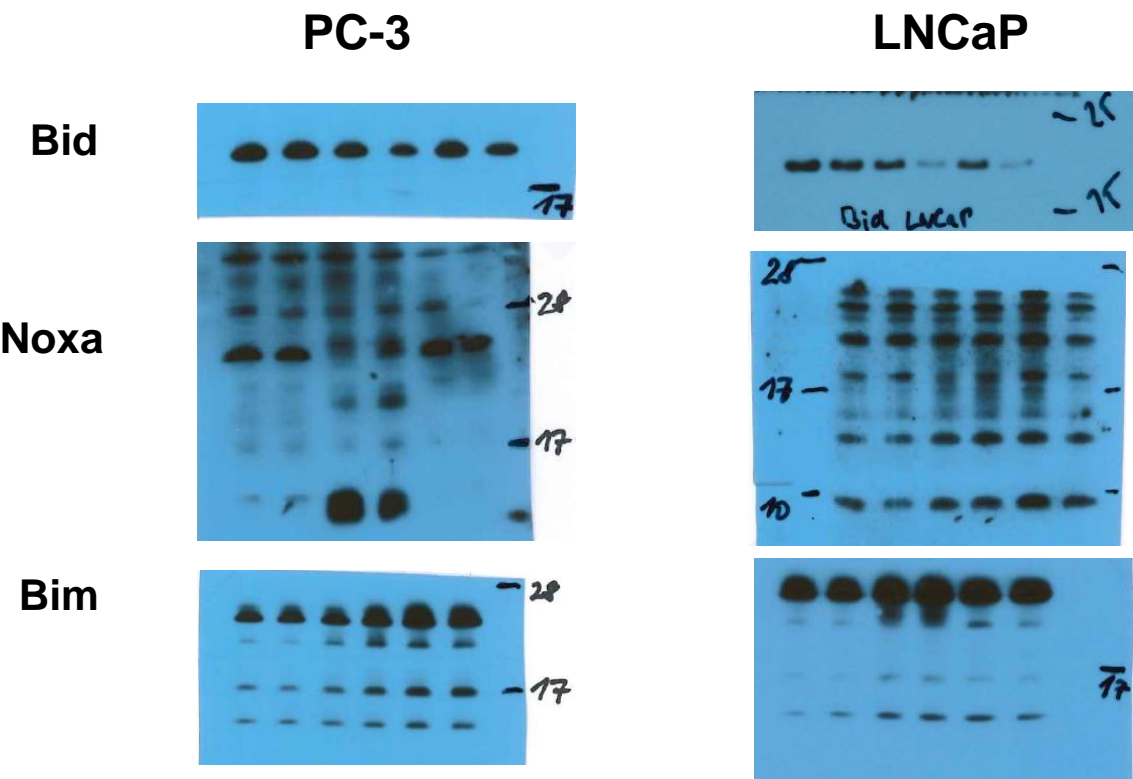

S3

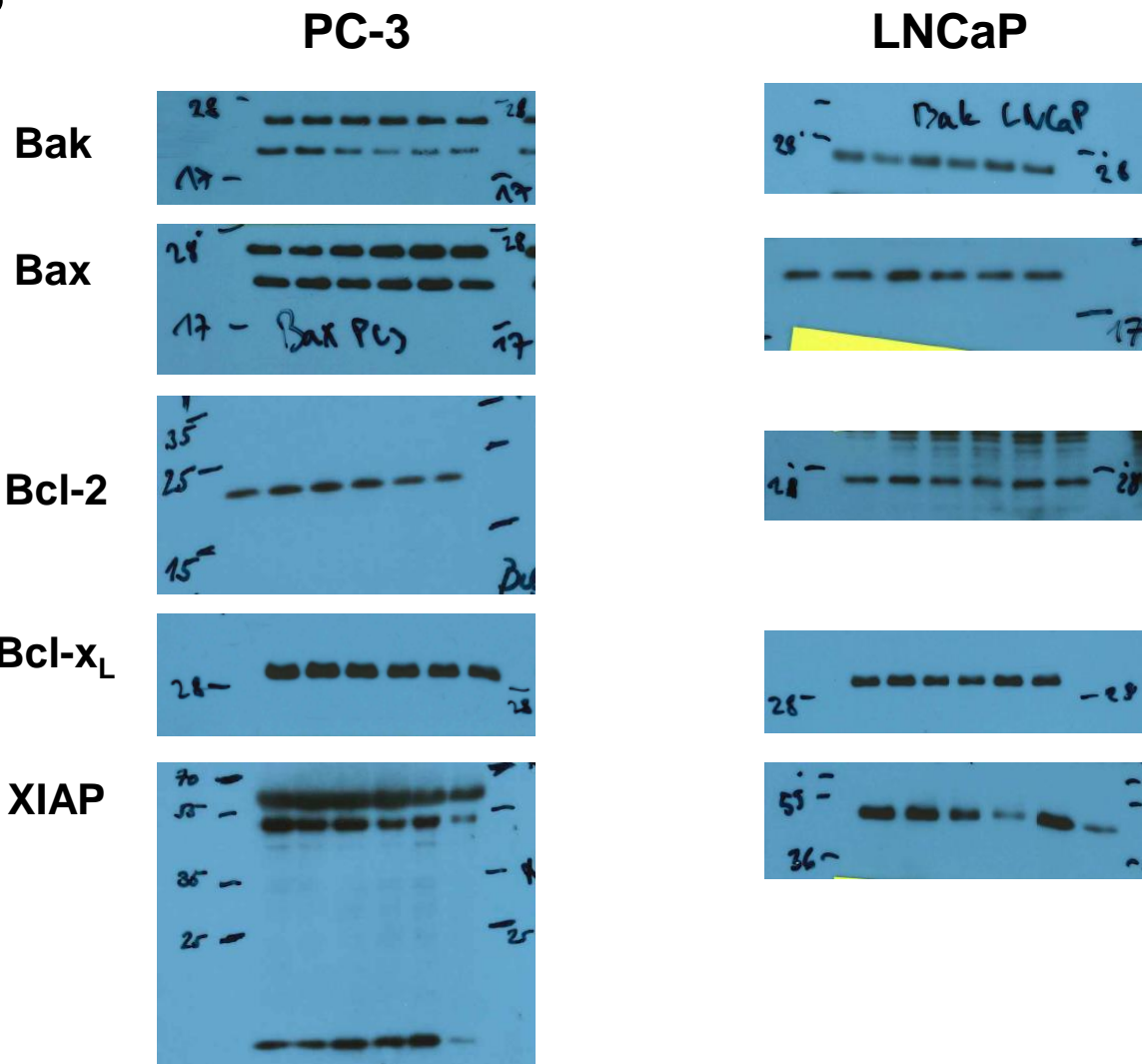

S6

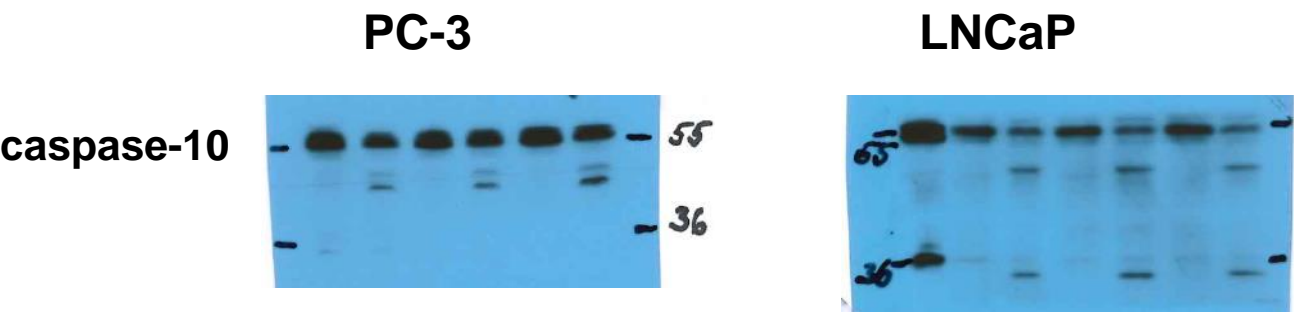

Supplement: S9 Fig — (PDF) [file pone.0188584.s009.pdf]
