# Supplementary material for: Cisplatin or LA-12 enhance killing effects of TRAIL in prostate cancer cells through Bid-dependent stimulation of mitochondrial apoptotic pathway but not caspase-10
Source: PLoS One. 2017 Nov 28;12(11):e0188584. doi: 10.1371/journal.pone.0188584 (PMC5705153; doi:10.1371/journal.pone.0188584)
Supplement: S10 Fig — (PDF) [file pone.0188584.s010.pdf]

Supplementary Material and Methods

Cell culture

Human colon adenocarcinoma cells HCT116 (obtained from prof. B. Vogelstein) were cultured in McCoy's 5A-modified medium (Gibco, Thermo Fisher Scientific) supplemented with penicillin (100 U/ml) and streptomycin (0.1 mg/ml) (both Duchefa Biochemie B. V.) and 10% fetal bovine serum (Gibco, Thermo Fisher Scientific). All cells were cultured and treated as specified in Material and Method section. In addition, calpain inhibitor - calpeptin was used (10 μM in DMSO, #03-34-0051, Calbiochem).

Immunoblotting analysis

In addition to the information specified in Material and Method section, immunodetection was carried out with polyclonal rabbit anti-Bax antibody produced by immunizing animals with a synthetic peptide corresponding to the amino-terminal residues of human Bax (1:1000, #2772, Cell Signaling Technology).

FLICA caspase-8 activity

The cells were harvested by trypsinization and stained in fresh culture media with FAM-FLICA (1:30, FAM-FLICA Caspase-8 kit, AbD Serotec, BioRad, USA) according to manufacturer's instructions. The percentage of cells with active caspase-8 was assessed by flow cytometry (FACS Verse) and analyzed by BD FACSuite v1.0.5 (both Becton Dickinson). Minimum of 10000 cells excluding debris and doublets were subjected to analysis.

Fluorimetric detection of caspase-9 activity

The cells were lysed in lysis buffer (50 mM HEPES, 5 mM CHAPS, 5 mM DTT, 1 μM aprotinin; Sigma–Aldrich) on ice for 20 min and then centrifuged at 15000 g for 15 min at 4 °C. The proteins acquired (equal concentrations) were incubated with caspase-9 substrate (Ac-LEHD-AFC, ALX-260-116-M005; Alexis Biochemicals Corporation, Lausen, Switzerland) overnight in assay buffer (20 mM HEPES, 2.5 mM CHAPS, 5 mM DTT, 2 mM EDTA) at 37

°C. Fluorescence was measured using Fluostar Galaxy fluorometer (BMG Labtechnologies; Offenburg, Germany).

CyQuant – Cell proliferation assay

Following the treatment in 96 well plate, the medium was aspirated and cells were incubated with the dye binding solution from the CyQUANT® NF Cell Proliferation Assay Kit (Invitrogen) according to the manufactures protocol. Finally, fluorescence was measured in a microplate reader FLUOSTAR Galaxy (BMG Labtechnologies GmbH, Offenburg, Germany).
